# Unimolecular Kinetics of Stabilized CH_3_CHOO Criegee Intermediates: *syn*-CH_3_CHOO Decomposition and *anti*-CH_3_CHOO Isomerization

**DOI:** 10.1021/acs.jpca.2c05461

**Published:** 2022-09-23

**Authors:** Callum Robinson, Lavinia Onel, James Newman, Rachel Lade, Kendrew Au, Leonid Sheps, Dwayne E. Heard, Paul W. Seakins, Mark A. Blitz, Daniel Stone

**Affiliations:** † School of Chemistry, University of Leeds, Woodhouse Lane, Leeds LS2 9JT, U.K.; ‡ Combustion Research Facility, Sandia National Laboratories, Livermore, California 94551, United States; § National Centre for Atmospheric Science, School of Chemistry, University of Leeds, Woodhouse Lane, Leeds LS2 9JT, U.K.

## Abstract

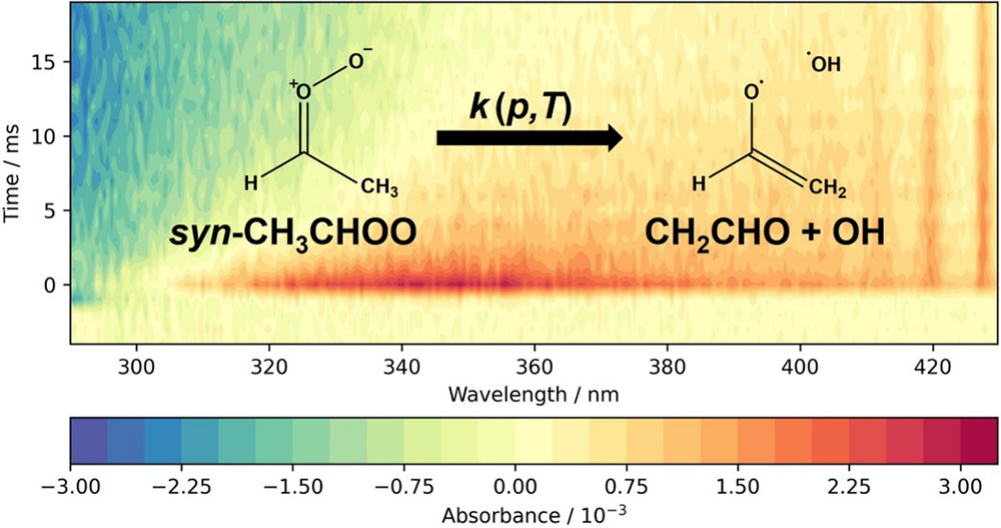

The kinetics of the unimolecular decomposition of the stabilized Criegee intermediate *syn*-CH_3_CHOO has been investigated at temperatures between 297 and 331 K and pressures between 12 and 300 Torr using laser flash photolysis of CH_3_CHI_2_/O_2_/N_2_ gas mixtures coupled with time-resolved broadband UV absorption spectroscopy. Fits to experimental results using the Master Equation Solver for Multi-Energy well Reactions (MESMER) indicate that the barrier height to decomposition is 67.2 ± 1.3 kJ mol^–1^ and that there is a strong tunneling component to the decomposition reaction under atmospheric conditions. At 298 K and 760 Torr, MESMER simulations indicate a rate coefficient of 150_–81_^+176^ s^–1^ when tunneling effects are included but only 5_–2_^+3^ s^–1^ when tunneling is not considered in the model. MESMER simulations were also performed for the unimolecular isomerization of the stabilized Criegee intermediate *anti*-CH_3_CHOO to methyldioxirane, indicating a rate coefficient of 54_–21_^+34^ s^–1^ at 298 K and 760 Torr, which is not impacted by tunneling effects. Expressions to describe the unimolecular kinetics of *syn*- and *anti*-CH_3_CHOO are provided for use in atmospheric models, and atmospheric implications are discussed.

## Introduction

Criegee intermediates (R_2_COO) are zwitterionic species produced in the atmosphere during the ozonolysis of unsaturated hydrocarbons and play a number of key roles in atmospheric oxidation processes. The high exothermicity (∼250 kJ mol^–1^)^1^ of ozonolysis reactions leads to the production of nascent excited Criegee intermediates with high internal energy which facilitates the production of atmospheric oxidants including the hydroxyl radical (OH), the hydroperoxy radical (HO_2_), and other peroxy radicals (RO_2_). Such processes have been recognized as important sources of OH, the primary oxidizing agent in the atmosphere, particularly in winter and at night when photolytic routes to OH production are limited by low or zero solar intensity^2,3^ but are in competition with collisional stabilization, which leads to the production of stabilized Criegee intermediates (SCIs). Once stabilized, SCIs can undergo bimolecular reactions with water and water dimers and can act as atmospheric oxidants in bimolecular reactions with species including SO_2_, NO_2_, and organic acids.^4−8^ There is also growing recognition that unimolecular SCI decomposition can be a significant loss mechanism for certain SCIs in the atmosphere, enhancing the production of OH from ozonolysis reactions beyond that achieved through the decomposition of nascent excited Criegee intermediates.^6−9^

**Figure 1:**
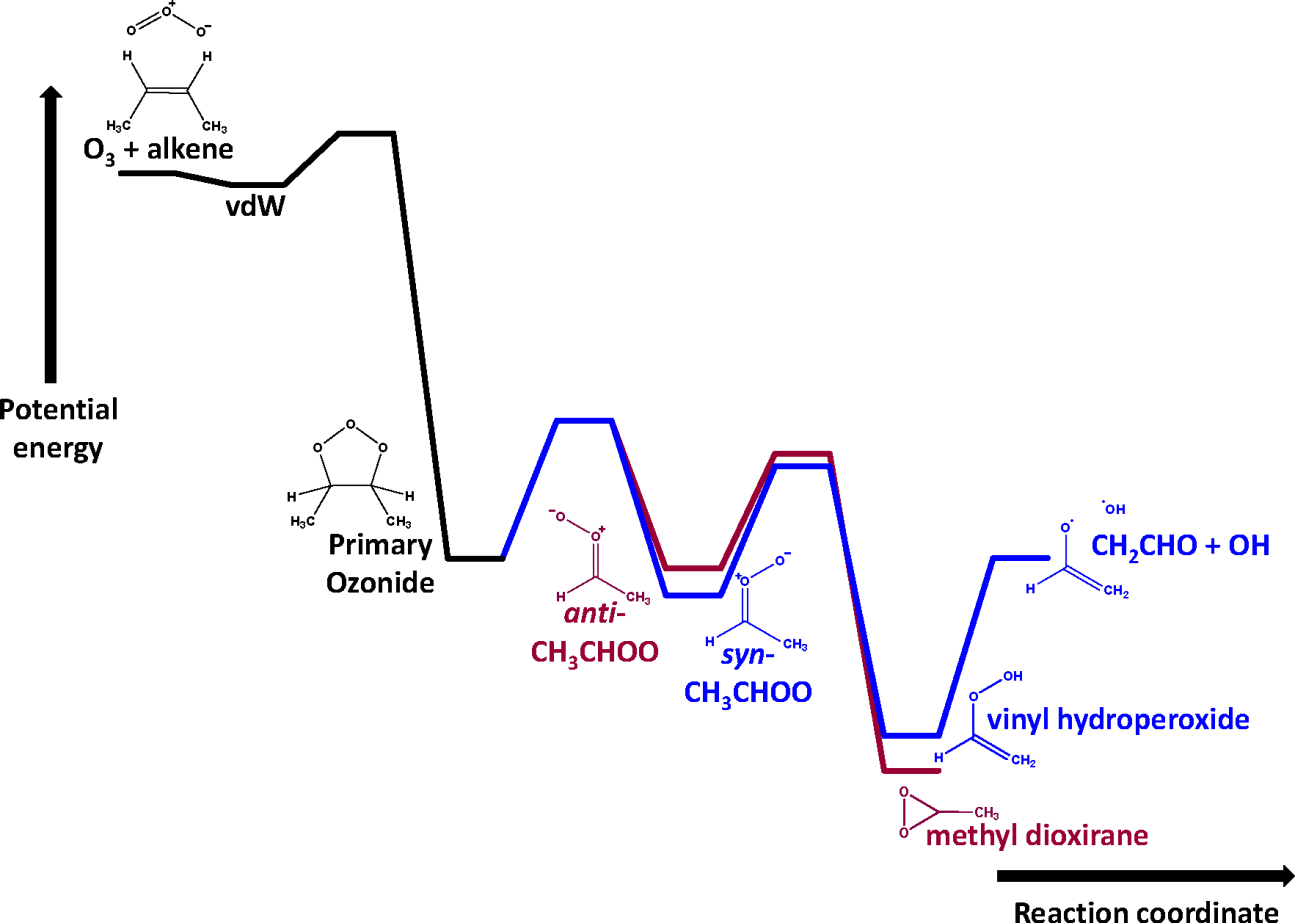
Typical potential energy surfaces describing the decomposition of *syn*-CH_3_CHOO and isomerization of *anti*-CH_3_CHOO (not to scale). Structures and labels refer to the species in the wells; vdW = van der Waals complex.

The Criegee intermediate CH_3_CHOO exists as two conformers: *syn*-CH_3_CHOO, in which the methyl group is *syn* to the terminal oxygen, and *anti*-CH_3_CHOO, in which the methyl group is *anti* to the terminal oxygen. Interconversion between the two conformers is precluded under atmospheric conditions by a significant barrier of ∼160 kJ mol^–1^ because of the double-bond character of the C–O bond resulting from the zwitterionic nature of Criegee intermediates,^10,11^ with the conformers displaying differences in spectra and reactivity.^11,12^ For *syn*-CH_3_CHOO, and other Criegee intermediates with α hydrogen atoms *syn* to the COO Criegee group, decomposition is facilitated by a 1,4-H transfer of the α-H atom to the terminal oxygen atom of the COO group, resulting in a rapid process that is expected to dominate the atmospheric chemistry of such SCIs.^7−9,13^ The 1,4-H transfer in *syn*-CH_3_CHOO leads to the production of vinyl hydroperoxide (CH_2_CHOOH, VHP) which can undergo decomposition to OH radicals and vinoxy radicals (CH_2_CHO) (R1).^7,13^ Other unimolecular pathways such as 1,3-cyclization of the COO group are expected to be slower than the 1,4-H transfer for *syn*-CH_3_CHOO.^13^ For *anti*-CH_3_CHOO, the 1,4-H transfer mechanism is limited by the restricted rotation around the C–O bond, and instead 1,3-cyclization of the COO Criegee group leads to the production of methyldioxirane (CH_3_CHO_2_) (R2).^7,13^ However, this process is expected to be relatively slow compared to the bimolecular reactions of *anti*-CH_3_CHOO.
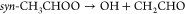
R1
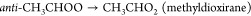
R2Figure 1 shows the typical potential energy surface summarizing the key features in the atmospheric formation and unimolecular chemistry of CH_3_CHOO Criegee intermediates.^4^ The kinetics of *syn*-CH_3_CHOO decomposition (R1) are key to determining the atmospheric fate and impact of *syn*-CH_3_CHOO and potentially other substituted SCIs with α-H atoms *syn* to the Criegee group, while the atmospheric losses of SCIs such as *anti*-CH_3_CHOO are expected to be dominated by bimolecular reactions, particularly the reactions with water vapor and water dimers.^7,13^

Studies of ozonolysis reactions in flow reactors and atmospheric simulation chambers have enabled estimates of the unimolecular kinetics of the stabilized Criegee intermediate CH_3_CHOO.^14−18^ However, such studies are typically unable to identify conformer-specific behavior and are subject to significant uncertainties because of the use of indirect measurements to infer the chemistry of Criegee intermediates. A number of such indirect studies have been used to approximate non-conformer-specific decomposition kinetics for CH_3_CHOO on the basis of relative rate arguments, giving rate coefficients ranging over several orders of magnitude (∼10^–3^ to ∼10^2^ s^–1^) at room temperature and atmospheric pressure,^14−17^ while the first absolute, but indirect, measurements reported a value of 76 s^–1^ with an accuracy within a factor of 3.^18^

Conformer-specific kinetics for *syn*-CH_3_CHOO decomposition have been reported following observations of OH formation in the ozonolysis of propene and *trans*-but-2-ene, with results for *k*_1_ ranging between 3 and 30 s^–1^ at ∼2.6 Torr and 293 K.^19^ Relative rate studies of CH_3_CHOO in ozonolysis experiments at ambient temperature and atmospheric pressure, involving reactions of CH_3_CHOO conformers with SO_2_ and measurements of either H_2_SO_4_ products^16^ or SO_2_,^17^ have also been used to estimate conformer-specific kinetics for *syn*- and *anti*-CH_3_CHOO. These studies indicate values for *k*_1_ of 86 ± 13 s^–1^ ^16^ and 310 ± 290 s^–1^,^17^ and a value of 38 ± 24 s^–1^ for *k*_2_,^16^ using current IUPAC recommendations^20^ for the kinetics of reactions of CH_3_CHOO conformers with SO_2_. However, these values are associated with large uncertainties, and the results are potentially affected by differences in pressure between the ozonolysis experiments and those performed to measure the kinetics of CH_3_CHOO + SO_2_.

The potential energy surfaces and kinetics for reactions R1 and R2 have also been studied by a number of groups using theoretical methods.^10,13,21−27^ The mechanism for (R1) is expected to proceed via 1,4-H atom transfer from the methyl substituent to the terminal oxygen atom in a cyclic five-membered transition state, while that for (R2) involves bending of the O–O bond and 1,3-cyclization of the COO Criegee group. Table 1 summarizes the methods used, calculated barriers, and the predicted kinetics. Despite a higher barrier, the predicted rate coefficients for *syn*-CH_3_CHOO (*k*_1_) are larger than those for *anti*-CH_3_CHOO (*k*_2_) in most studies because of expected effects of quantum mechanical tunneling. For *syn*-CH_3_CHOO, the availability of the 1,4-H transfer pathway facilitates quantum mechanical tunneling through the reaction barrier, leading to a significant enhancement in the rate of reaction which is not possible for *anti*-CH_3_CHOO since pathways involving H atom transfer are inaccessible because of the restricted rotation around the C–O bond. The impact of quantum mechanical tunneling on the decomposition of *syn*-CH_3_CHOO has been investigated in a series of experiments involving vibrational activation of jet-cooled *syn*-CH_3_CHOO, generated via photolysis of CH_3_CHI_2_/O_2_, and detection of the OH radical decomposition product.^9,22,23,28−31^ Activation of specific vibrational modes in *syn*-CH_3_CHOO using tunable infrared laser excitation, while remaining below the energy barrier to decomposition, has demonstrated that there is a significant tunneling component to the reaction, with a combination of experimental results and application of theory leading to an expected rate coefficient of 122 s^–1^ at 298 K and atmospheric pressure.^23^ Experiments using *syn*-CD_3_CHOO have also indicated that the decomposition of *syn*-CH_3_CHOO proceeds predominantly by a tunneling mechanism, with a kinetic isotope effect of over 50.^32^

**Table 1:** Summary of Theoretical Studies of Reactions R1 and R2^a^

*T*/K	*p*/Torr	*k*_1_/s^–1^	barrier height (R1)/kJ mol^–1^	*k*_2_/s^–1^	barrier height (R2)/kJ mol^–1^	methods	ref
298	∞	24.2	74.9	67.2	64.4	MCG3//QC1SD/MG3	Kuwata et al.^10^
						RRKM, VTST & Eckart tunneling model	
298	∞	209	69.5	57.6	64.4	CCSD(T)/aug-cc-pVTZ//B3LYP/aug-cc-pVTZ	Nguyen et al.^21^
						RRKM & Eckart tunnelling model	
298	760	166	71.5			CCSD(T) cc-pVTZ & CCSD(T)-F12 CBS	Fang et al.^22^
						RRKM & Eckart tunneling model	
298	760	122	71.5			CCSD(T) cc-pVTZ & CCSD(T)-F12 CBS	Fang et al.^23^
						RRKM & adjusted Eckart tunneling model	
298	∞	328	71.2	55.4	65.4	W3X-L//CCSD(T)-F12a/DZ-F12	Long et al.^24^
						MP-CVT SCT	
298	760	124	72			mHEAT-345(Q)	Nguyen et al.^25^
						SCTST & VPT2	
298	∞	182	69.8	72.5	64.4	QCISD(T)/CBS//B3LYP/6-311+G(2d,2p)	Vin & Takahashi^26^
						SCTST & VPT2	
298	∞	136	70.3	53	65.7	CCSD(T)/aug-cc-pVTZ//M06-ZX	Vereecken et al.^13^
						MC-CTST & Eckart tunneling model	
291	∞	137	71.1			MP2/aug-cc-pVTZ & CCSD(T)/aug-cc-pVTZ	Burd et al.^27^
						1D-SCTST	
291	∞	155	71.1			MP2/aug-cc-pVTZ & CCSD(T)/aug-cc-pVTZ	Burd et al.^27^
						FD-SCTST (MP2 x matrix)	
291	∞	136	71.1			MP2/aug-cc-pVTZ & CCSD(T)/aug-cc-pVTZ	Burd et al.^27^
						FD-SCTST (B3LYP x matrix)	

a Temperatures and pressures are given for the conditions at which the rate coefficients are calculated.

Direct measurements of the kinetics of *syn*-CH_3_CHOO decomposition have been made at 298 K at pressures between 10 and 100 Torr, with CH_3_CHOO produced following photolysis of CH_3_CHI_2_/O_2_/Ar gas mixtures at 248 nm.^33^ The kinetics for *syn*-CH_3_CHOO were determined through detection of OH radicals by high repetition rate (10 kHz) laser-induced fluorescence at 282 nm, based on the assumption that any OH in the system is formed exclusively from decomposition of the *syn*-conformer. Analysis of the temporal profiles for OH required not only consideration of the kinetics for the decomposition of the Criegee intermediate but also its reactions with other species in the reaction mixture, including the CH_3_CHI_2_ precursor, IO radicals, and its self-reaction, as well as the kinetics of the processes leading to the removal of OH from the system. Knowledge of the initial concentrations of *syn*-CH_3_CHOO was also required and was estimated to be on the order of 10^12^–10^13^ cm^–3^ from the laser fluence, the CH_3_CHI_2_ precursor concentration, the yield of CH_3_CHOO from the reaction between CH_3_CHI and O_2_, which was assumed to be 0.9 for all pressures investigated based on previous measurements at 4 Torr,^11^ and the ratio of *syn*:*anti* conformers, which was assumed to be 7:3.^12^ The impact of wall losses and diffusion of *syn*-CH_3_CHOO were assumed to be negligible. Determination of the decomposition kinetics using measurements of OH thus requires a complex analysis which necessitates a number of assumptions, although application of the approach to measure the kinetics of *syn*-CH_3_CHOO + SO_2_ gave similar results to others reported in the literature.^11,12,34^ The results for the decomposition kinetics of *syn*-CH_3_CHOO indicated a rate coefficient of 182 ± 66 s^–1^ between 25 and 100 Torr, with no significant pressure dependence in this range, although a lower value of ∼70 s^–1^ was obtained at 10 Torr.^33^

Experiments using time-resolved UV absorption spectroscopy have also been performed to make direct measurements of the decomposition kinetics of *syn*-CH_3_CHOO, enabling investigation of the pressure dependence between 100 and 700 Torr at 298 K and of the temperature dependence between 278 and 318 K at a pressure of 300 Torr.^35^ Photolysis of CH_3_CHI_2_/O_2_/N_2_ at 248 nm was used to generate CH_3_CHOO, with the absorption monitored at 340 nm. While both *syn*- and *anti*-CH_3_CHOO display significant absorption at 340 nm, high concentrations of water vapor were added to ensure the rapid removal of *anti*-CH_3_CHOO. The change in absorption at 340 nm was thus dominated by changes in the concentration of *syn*-CH_3_CHOO, with minor contributions from CH_3_CHI_2_, which are approximately constant on the time scale of the experiment, and IO. Compared to the experiments involving detection of OH,^33^ direct monitoring of *syn*-CH_3_CHOO simplifies the analysis, although determination of the decomposition kinetics still required consideration of the *syn*-CH_3_CHOO self-reaction, which was assumed to be negligible for initial concentrations on the order of 10^11^ cm^–3^, and of the reactions involving CH_3_CHI_2_, IO, and, for these experiments, water vapor. The reaction of *syn*-CH_3_CHOO with its di-iodo precursor was demonstrated to be significant, with similar effects observed at high temperatures in our previous investigation of the decomposition kinetics of CH_2_OO.^36^ The effects of physical losses such as diffusion and wall loss were estimated on the basis of measurements for the CH_2_OO Criegee intermediate under similar conditions and were highlighted as being potentially underestimated in the previous work^33^ involving detection of OH. Results indicated a weak pressure dependence in the decomposition kinetics of *syn*-CH_3_CHOO at 298 K, with the rate coefficient varying from ∼120 s^–1^ at 100 Torr to ∼170 s^–1^ at 700 Torr, and an increase in the rate coefficient at 300 Torr from 67 ± 15 s^–1^ at 278 K to 146 ± 31 s^–1^ at 298 K and 288 ± 81 s^–1^ at 318 K.^35^

There are thus a range of estimates for CH_3_CHOO Criegee intermediate decomposition kinetics obtained from various theoretical and experimental approaches, with significant uncertainties remaining. However, there is agreement within the literature that quantum mechanical tunneling plays an important role in the decomposition of *syn*-CH_3_CHOO under ambient conditions and that the reaction is likely to dominate its atmospheric chemistry, with potentially significant consequences for tropospheric OH production.

In this work we report a direct investigation of the conformer-specific kinetics of *syn*-CH_3_CHOO decomposition. Experiments were conducted at pressures between 12 and 300 Torr and temperatures between 297 and 331 K using flash photolysis of CH_3_CHI_2_/O_2_/N_2_, coupled with time-resolved broadband UV absorption spectroscopy. Experimental results are supported by Master equation calculations performed using the Master Equation Solver for Multi-Energy well Reactions (MESMER) to provide a full parametrization of the kinetics as a function of temperature and pressure suitable for inclusion in atmospheric models.

## Experimental Section

The decomposition kinetics of *syn*-CH_3_CHOO were studied using flash photolysis of CH_3_CHI_2_/O_2_/N_2_ gas mixtures, coupled with broadband UV absorption spectroscopy. The experimental apparatus has been described in detail elsewhere;^37−40^ therefore, only a brief description is given here.

Precursor and bath gases were mixed in a gas manifold at known flow rates controlled by calibrated mass flow controllers (MKS Instruments), with CH_3_CHI_2_ entrained into the flow by passing a known flow of N_2_ through a bubbler containing liquid CH_3_CHI_2_ held at a constant temperature in an ice bath. The precursor gas mixture was passed into a jacketed Pyrex reaction cell (100 cm in length, 3 cm internal diameter) which was sealed with fused silica windows at each end. The total flow rate through the reaction cell was maintained at 4000 standard cm^3^ per minute (sccm) at 100 Torr and adjusted accordingly with pressure to maintain a constant residence time in the cell of ∼6 s. The total pressure in the cell was measured by a capacitance manometer (MKS Instruments) and controlled by a rotary pump (EM2, Edwards) by throttling the exit to the reaction cell. The temperature of the gas mixture was maintained by flowing liquid from a recirculating thermostatting unit (Huber Unistat 360) through the jacket surrounding the cell and calibrated through measurements of the temperature in the cell made by a K-type thermocouple placed at a series of positions along the length of the cell in separate experiments using flowing N_2_ gas under otherwise identical conditions.^40^

Chemistry in the cell was initiated by an excimer laser (KrF, Lambda-Physik CompEx 210) operating at a wavelength of λ = 248 nm, which was aligned along the length of the reaction cell using a dichroic turning mirror (Edmund Optics). The timing of the photolysis laser was controlled by a delay generator (SRS DG535) with a pulse repetition rate of 0.15 Hz such that a fresh gas mixture was photolyzed on each pulse. The typical laser fluence was ∼25 mJ cm^–2^, giving [*syn*-CH_3_CHOO]_0_ on the order of ∼10^11^–10^12^ cm^–3^.

Absorption of UV/vis radiation by species within the cell was monitored using a laser-driven light source (LDLS, Energetiq EQ-99X), which provides ∼10 mW cm^–2^ of light at wavelengths between 200 and 800 nm with near constant radiance across the spectral range. Output from the lamp was directed onto an off-axis parabolic mirror (ThorLabs) to collimate the beam. The probe light was aligned in a seven-pass arrangement described previously, resulting in a total effective path length of 443 ± 21 cm.^37−39^ The beam exiting the cell was passed through a sharp cut-on filter (248 nm RazorEdge ultrasteep long-pass edge filter, Semrock) to minimize the impacts of scattered excimer light and focused onto a fiber optic via a fiber launcher (Elliot Scientific).

For experiments at *T* = 297 K, the output from the fiber optic was directed onto a spectrograph (CP140-103 Imaging Spectrograph, Horiba) and imaged onto a line-scan charge-coupled device (CCD) detector (S7030-1006 FFT, Hamamatsu), giving a spectral resolution (FWHM) of 1.5 nm and a time resolution of 1 ms achieved by transfer of data from the CCD to a PC for analysis in real time at 1 ms intervals.^37,38^

For experiments at *T* > 297 K, the output from the fiber optic was directed through a 25 μm slit onto a spectrograph equipped with a diffraction grating of 300 grooves/mm and imaged onto an integrated thermoelectrically cooled charge-coupled device (CCD) detector (FER-SCI-1024BRX, Princeton Instruments) with a spectral resolution (FWHM) of 1 nm and a variable time resolution on the order of hundreds of microseconds. The improved time resolution for experiments at *T* > 297 K was necessary because of the more rapid chemistry occurring at such temperatures and required use of charge transfer from an illuminated region of the CCD (1024 × 10 pixels) to an optically masked storage region (1024 × 265 pixels) on the CCD prior to transfer to the PC for analysis. Charge transfer on the CCD, which can take place more rapidly than the communication between the CCD and the PC, requires the illumination of multiple rows on the CCD and results in an instrument response function which is also applied to the model used to obtain kinetic data during analysis (further details are given in the Supporting Information).^39^

For experiments at all temperatures, the CCDs provide a series of sequential, time-resolved broadband transmission spectra before, during, and after photolysis. Wavelength calibration was performed via measurements of the well-known Hg emission spectrum from a low pressure Hg Pen-Ray lamp (Oriel). Timing of the CCD cameras was controlled by the same delay generator used to control the firing of the excimer laser. Intensity data recorded by the cameras were typically averaged for 100–400 photolysis shots and were transferred to a PC for analysis.

Experiments were performed in N_2_ (BOC oxygen free, 99.998%) at temperatures between 297 and 331 K and pressures between 12 and 300 Torr. Concentrations of CH_3_CHI_2_ (Sigma-Aldrich, 98%) were varied in the range 4.33 × 10^12^–2.80 × 10^14^ cm^–3^ to enable characterization of the kinetics of CH_3_CHI_2_ + CH_3_CHOO, while also ensuring low concentrations of photolysis products to minimize the effects of possible Criegee–Criegee and Criegee–radical chemistry. Concentrations of O_2_ (BOC, 99.5%) were varied between 9.15 × 10^16^ and 5.89 × 10^17^ cm^–3^, while maintaining the requirement for rapid production of CH_3_CHOO following photolysis of CH_3_CHI_2_. Gases and chemicals were used as supplied.

## Results

Absorbance spectra were determined at each time point during the course of the reaction from the measured transmission spectra using the Beer–Lambert law eq 1:
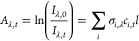
1where *A*_λ*,t*_ is the total absorbance at wavelength λ and time *t*, *I*_λ*,*__0_ is the average pre-photolysis light intensity at wavelength λ, *I*_λ*,t*_ is the post-photolysis light intensity at wavelength λ and time *t*, σ_*i,*λ_ is absorption cross section of species *i* at wavelength λ, *c*_*i,t*_ is the concentration of species *i* at time *t*, and *l* is the effective path length, which has a value of 443 ± 21 cm.

**Figure 2:**
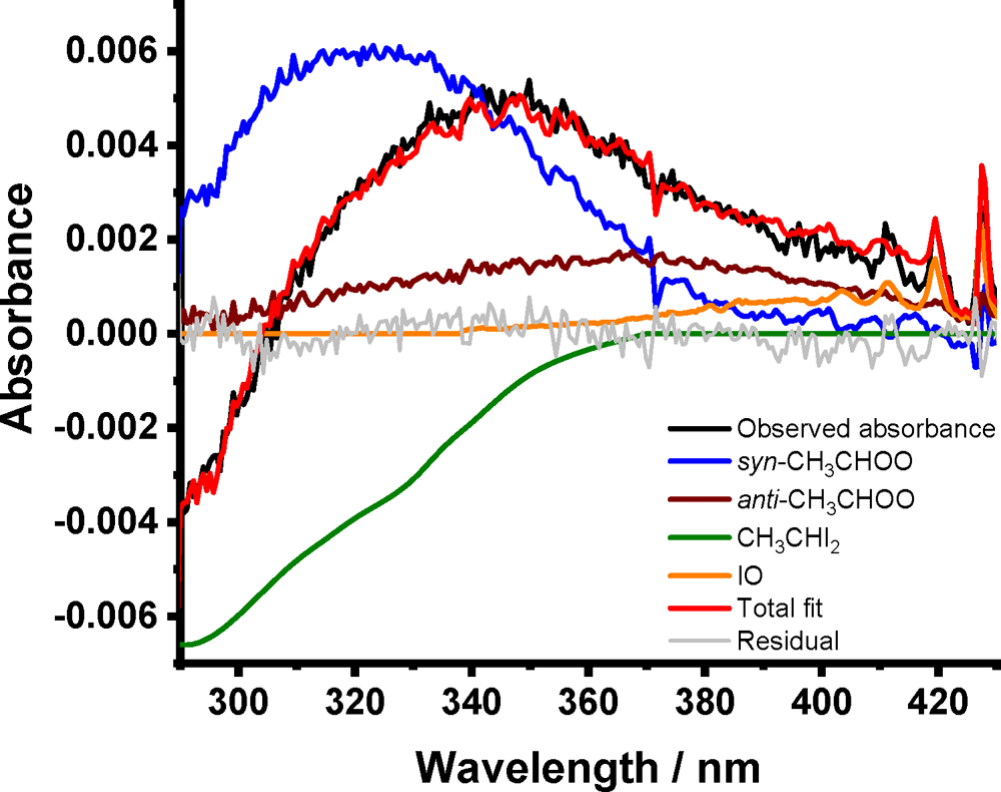
Observed absorbance (black), total fit (red), and the individual contributions from *syn*-CH_3_CHOO (blue), *anti*-CH_3_CHOO (purple), CH_3_CHI_2_ (green), and IO (orange) determined by fitting reference spectra to the observed absorbance using eq 1. For these data, *T* = 297 K, *p* = 12 Torr, *t* = 1.0 ms, and [CH_3_CHI_2_] = 2.6 × 10^13^ cm^–3^. The fit to the observed absorbance for these data gave Δ[CH_3_CHI_2_] = 3.97 × 10^12^ cm^–3^, [*syn*-CH_3_CHOO] = 1.14 × 10^12^ cm^–3^, [*anti*-CH_3_CHOO] = 3.00 × 10^11^ cm^–3^, and [IO] = 1.95 × 10^11^ cm^–3^.

**Figure 3:**
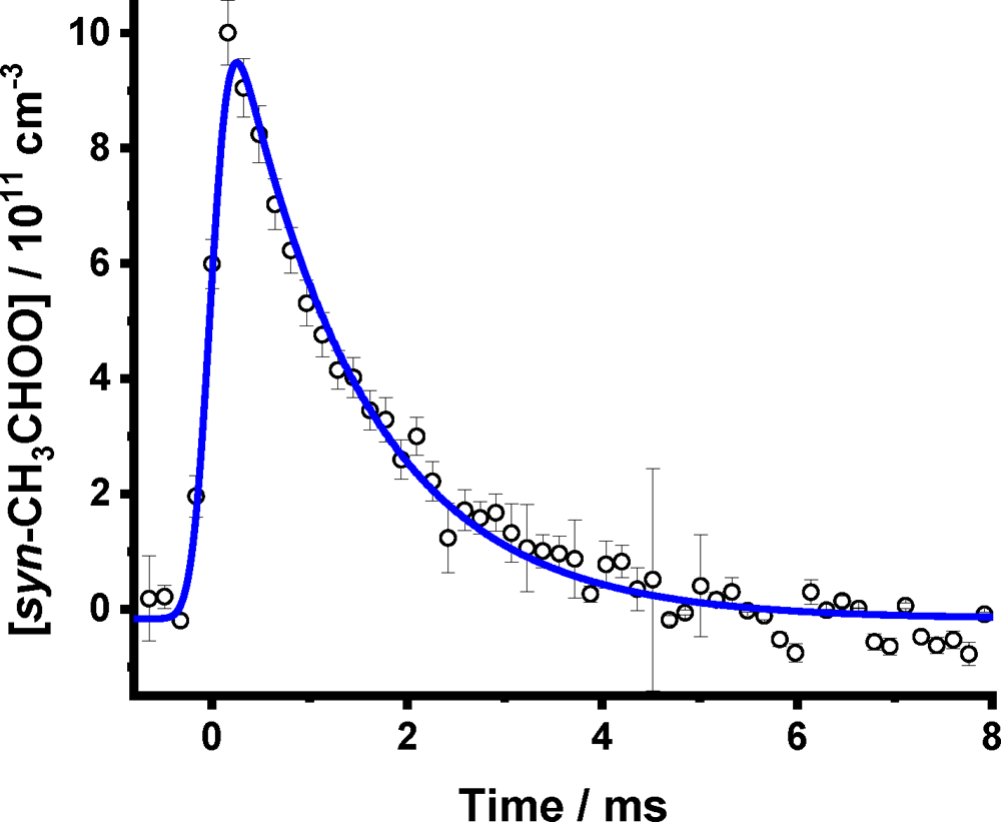
Observed concentration–time profile for [*syn*-CH_3_CHOO] (open circles). For these data, *T* = 318 K, *p* = 160 Torr, and [CH_3_CHI_2_] = 2.4 × 10^13^ cm^–3^. Uncertainties in the *syn*-CH_3_CHOO concentrations are given by the uncertainties in the fits to the observed absorbance at each time point combined with the uncertainty in the effective path length. The fit to eq 3 (coupled with the instrument response function as detailed in the Supporting Information) (solid line) gave [*syn*-CH_3_CHOO]_0_ = (1.24 ± 0.08) × 10^12^ cm^–3^ and *k*′ = 756 ± 79 s^–1^ (with the instrument response parameters *w* = (1.52 ± 0.04) × 10^–4^ s and *t*_c_ = −(8.9 ± 3.2) × 10^–6^ s). Errors are 1σ.

Reference absorption cross sections for CH_3_CHI_2_,^41^*syn*-CH_3_CHOO,^12^*anti*-CH_3_CHOO,^12^ and IO^42^ were least squares fit to the absorbance spectra to obtain concentrations for each species throughout the reaction. Figure 2 shows a typical fit to the post-photolysis absorbance, with typical concentration–time profiles shown in Figure 3. Experimental time scales and precursor concentrations were optimized to obtain kinetics for *syn*-CH_3_CHOO, which resulted in small contributions to the absorbance signal from *anti*-CH_3_CHOO because of lower yields of the *anti*-conformer from CH_3_CHI + O_2_^11,12,34^ and, presumably, more rapid reaction of the *anti*-conformer with the CH_3_CHI_2_ precursor. Experimental results reported in this work focus on the observations of *syn*-CH_3_CHOO, for which conditions were optimized.

The kinetics of *syn*-CH_3_CHOO removal from the system are controlled by its decomposition (R1), reaction with the CH_3_CHI_2_ precursor (R3), physical losses such diffusion out of the probe region and wall loss (R4), self-reaction (R5), and reaction with *anti*-CH_3_CHOO (R6) or iodine atoms (R7).
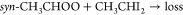
R3
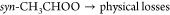
R4
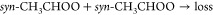
R5
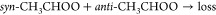
R6
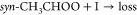
R7

The observed decays are also influenced by an instrument response function, details of which are given in the Supporting Information. Of the processes contributing to *syn*-CH_3_CHOO removal, the reaction with the precursor is pseudo-first-order because of the higher concentrations of CH_3_CHI_2_ compared to *syn*-CH_3_CHOO, decomposition is first-order, and physical losses can be approximated as first-order, while the *syn*-CH_3_CHOO self-reaction and reactions with *anti*-CH_3_CHOO or iodine atoms are second-order. Concentration–time profiles for *syn*-CH_3_CHOO can therefore be fit with a mixed first- and second-order kinetic model (eq 2), which can be coupled with the instrument response function where necessary (see the Supporting Information.
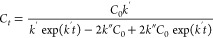
2where *C*_*t*_ is the concentration of *syn*-CH_3_CHOO at time *t*, *C*_0_ is the initial concentration of *syn*-CH_3_CHOO, *k*′ represents the sum of first-order (or pseudo-first-order) losses of *syn*-CH_3_CHOO, and *k*″ represents the sum of second-order losses of *syn*-CH_3_CHOO.

Fits to eq 2 were performed with *k*″ treated as a global parameter at each temperature and pressure and all other parameters treated locally. At 297 K, the fits gave an average value of *k*″ = (7.8 ± 3.0) × 10^–11^ cm^3^ s^–1^, with no significant dependence on pressure (see the Supporting Information). At temperatures above 297 K, fits to eq 2 were found to be insensitive to the second-order component, indicating minimal effects of Criegee–Criegee and Criegee–iodine reactions for the initial concentrations used in these experiments (see the Supporting Information for further details). Similar behavior was also observed in our previous study of CH_2_OO Criegee decomposition^36^ and in a previous study of *syn*-CH_3_CHOO decomposition.^35^ For data obtained at temperatures above 297 K, the *syn*-CH_3_CHOO profiles were thus analyzed with a model based on first-order loss kinetics (eq 3), which was coupled with the instrument response function where necessary as detailed in the Supporting Information.
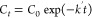
3where *C*_*t*_ is the concentration of *syn*-CH_3_CHOO at time *t*, *C*_0_ is the initial concentration of *syn*-CH_3_CHOO, and *k*′ is the rate coefficient describing the sum of first-order loss of *syn*-CH_3_CHOO.

**Figure 4:**
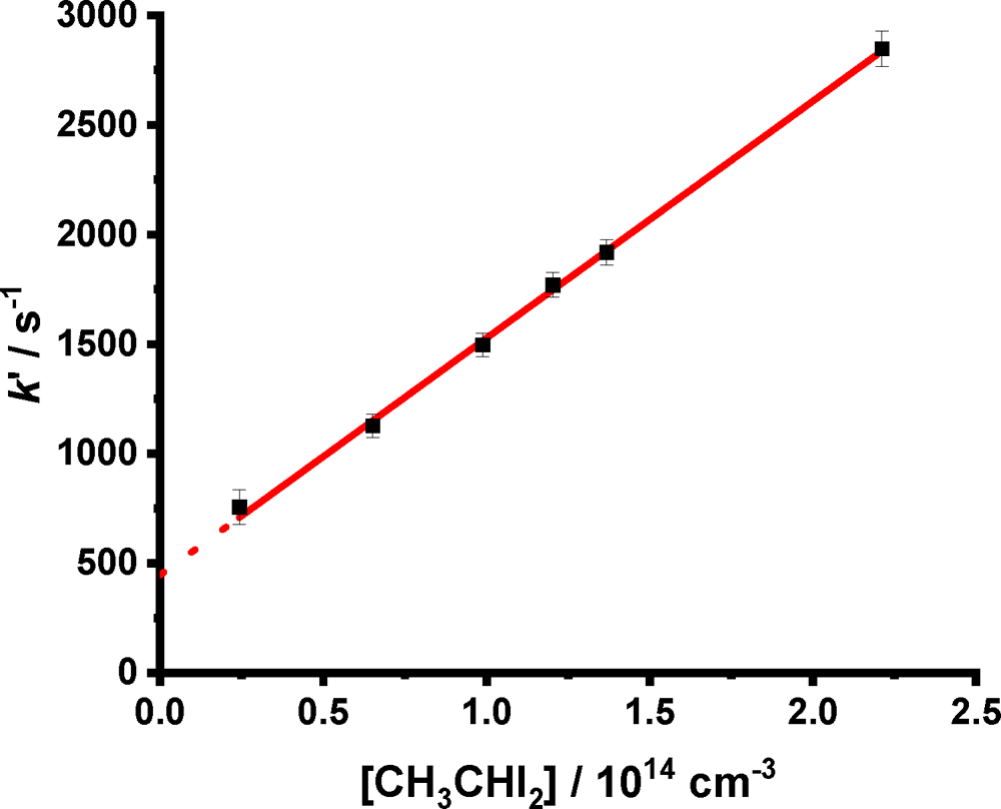
*k*′ against [CH_3_CHI_2_] at *T* = 318 K and *p* = 160 Torr (black points). The fit to the data (solid line) gave (*k*_1_ + *k*_4_) = 446 ± 115 s^–1^ (intercept) and *k*_3_ = (10.81 ± 0.98) × 10^–12^ cm^3^ s^–1^ (slope). Errors are 1σ.

For fits to eq 2 or 3, the observed first-order rate coefficient describing the loss of *syn*-CH_3_CHOO from the system, *k*′, is equal to *k*_1_ + *k*_3_[CH_3_CHI_2_] + *k*_4_, and a plot of *k*′ against the concentration of the CH_3_CHI_2_ precursor yields a slope equal to *k*_3_ and an intercept equal to *k*_1_ + *k*_4_. At each temperature and pressure investigated the concentration of CH_3_CHI_2_ was varied sufficiently to determine *k*_3_. Figure 4 shows an example plot of *k*′ against [CH_3_CHI_2_] used to determine (*k*_1_ + *k*_4_) and *k*_3_. Although there is some variability in determinations of *k*_3_, potentially because of uncertainties in [CH_3_CHI_2_], the gradients and intercepts of the plots of *k*′ against [CH_3_CHI_2_] are well-defined, and thus (*k*_1_ + *k*_4_) is well-defined. Results for *k*_3_ indicate no significant dependence on pressure and a temperature dependence described by *k*_3_ = (3.2 ± 0.7) × 10^–10^ exp((−1230 ± 70)/*T*) cm^3^ s^–1^, with a mean value of (5.1 ± 2.4) × 10^–12^ cm^3^ s^–1^ at 297 K (see the Supporting Information for further details).

Effects of physical losses were estimated from experiments in which the physical losses of the Criegee intermediate CH_2_OO were measured via photolysis of CH_2_I_2_/O_2_/N_2_ mixtures,^38^ in a similar manner to that described by Li et al.^35^ in their study of *syn*-CH_3_CHOO decomposition. Li et al. demonstrated that calculations of physical losses of *syn*-CH_3_CHOO via diffusion likely underestimate the total physical losses because of effects of turbulence and that measurements of CH_2_OO can be used to better estimate the total physical losses because the decomposition kinetics of CH_2_OO is slow.^36^ For pressures above 12 Torr, the rate coefficient describing physical losses obtained from measurements of CH_2_OO showed no significant dependence on pressure, with a mean value of 3.2 ± 1.7 s^–1^, while at a pressure of 12 Torr a value of 10.6 ± 5.9 s^–1^ was obtained. A similar value of 9 ± 6 s^–1^ was reported by Li et al.^35^ for a similar experimental setup, with no significant dependence on temperature in the range 278–318 K or pressure in the range 100–700 Torr. The decomposition kinetics for *syn*-CH_3_CHOO was subsequently obtained by subtracting the estimated rate coefficients for physical losses (*k*_4_) from the intercepts of the plots of *k*′ against the concentration of CH_3_CHI_2_ (equal to *k*_1_ + *k*_4_) to obtain rate coefficients for *syn*-CH_3_CHOO decomposition (*k*_1_). Physical losses were significantly lower than chemical losses through decomposition for all conditions (i.e., *k*_4_ ≪ *k*_1_). Further details are given in the Supporting Information.

**Figure 5:**
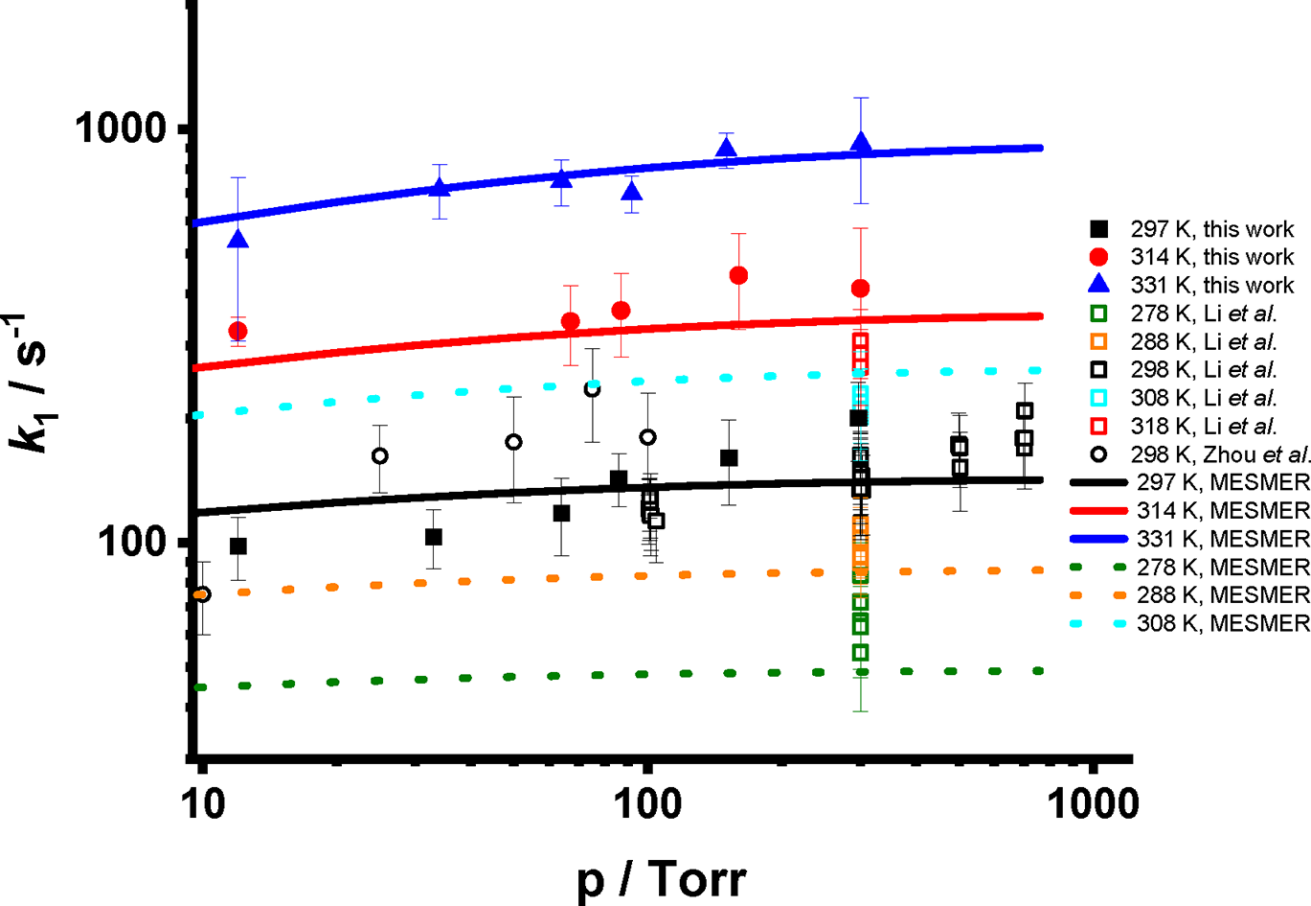
*k*_1_ as a function of temperature and pressure. Results from this work are shown by the solid symbols; those obtained by Zhou et al.^33^ and Li et al.^35^ are shown by the open symbols. MESMER simulations using the parameters obtained from fits to this work are shown by the solid lines, with simulations using the fitted parameters for conditions used in previous work shown by the dotted lines. Errors are 1σ.

Figure 5 shows the rate coefficients for *syn*-CH_3_CHOO decomposition, *k*_1_, as a function of temperature and pressure. A summary is given in Table 2. At 297 K, *k*_1_ varies from 98.1 ± 16.9 s^–1^ at 12 Torr to 200.6 ± 43.1 s^–1^ at 297 Torr, with results in agreement with previous measurements using photolysis of CH_3_CHI_2_/O_2_ to generate *syn*-CH_3_CHOO,^33,35^ although the results obtained by Zhou et al.,^33^ based on detection of OH, are systematically higher than those obtained in this work and by Li et al.,^35^ which both monitor *syn*-CH_3_CHOO directly. Results are also in good agreement with theoretical predictions at ∼298 K (Table 1) and measurements obtained at 300 Torr and 318 K by Li et al.^35^

**Table 2:** Summary of Experimental Conditions and Results (Errors Are 1σ)

*T*/K	*p*/Torr	(*k*_1_ + *k*_4_)/s^–1^	*k*_3_/10^–12^ cm^3^ s^–1^	*k*_1_/s^–1^
297	12	109 ± 16	4.82 ± 0.37	98 ± 17
297	33	107 ± 17	5.21 ± 0.44	103 ± 17
297	64	121 ± 25	5.54 ± 0.49	118 ± 25
297	86	146 ± 21	1.52 ± 1.12	143 ± 21
297	152	164 ± 37	4.76 ± 0.86	161 ± 37
297	297	204 ± 43	9.03 ± 2.98	201 ± 43
314	12	335 ± 26	4.83 ± 0.37	325 ± 27
314	67	346 ± 75	1.78 ± 0.59	343 ± 75
314	87	367 ± 83	3.85 ± 0.83	364 ± 83
314	160	446 ± 115	10.81 ± 0.98	443 ± 115
314	300	415 ± 163	12.02 ± 1.23	412 ± 163
331	12	546 ± 228	8.38 ± 2.46	535 ± 228
331	34	716 ± 108	9.23 ± 0.75	713 ± 108
331	64	752 ± 95	8.99 ± 0.62	749 ± 96
331	92	701 ± 72	8.76 ± 0.59	698 ± 72
331	150	893 ± 87	8.00 ± 0.71	890 ± 87
331	301	927 ± 265	4.13 ± 2.07	924 ± 265

### Master Equation Analysis

Master equation calculations were performed to rationalize the observed decomposition kinetics for *syn*-CH_3_CHOO, and to predict the isomerization kinetics for *anti*-CH_3_CHOO, using the Master Equation Solver for Multi-Energy well Reactions (MESMER), which has been described in detail in previous work.^36,40,43,44^ MESMER uses an energy-grained master equation in which the rovibrational energy states for reactants, transition states, intermediates, and products are partitioned into a number of grains that contain a defined number of states. Energy grains representing initial reactants are assigned populations based on a Boltzmann distribution, with grains representing other species assigned a population of zero. Changes in the population distribution among the grains can occur through collisional energy transfer via interactions with a thermal bath gas or through reactive transformation of one species to another. Transformations of one species to another are controlled by the microcanonical rate coefficients in the system, which are described by RRKM theory, while collisional energy transfer is described by an exponential down model in which the average energy transferred between grains on collision is determined by the parameter ⟨Δ*E*⟩_down_. In this work, ⟨Δ*E*⟩_down_ was assumed to be independent of temperature because of the relatively narrow range of temperatures investigated.

Pressure- and temperature-dependent rate coefficients for *k*_1_ were calculated in MESMER using a rigid rotor harmonic oscillator approximation, with the effects of quantum mechanical tunneling determined using the asymmetric Eckart tunneling model.^45^ Relevant energies, geometries, vibrational frequencies, and rotational constants were provided by the calculations reported by Vereecken et al.,^13^ which were performed at the CCSD(T)/aug-cc-pVTZ//M06-ZX level of theory. Collision parameters were obtained from the work of Long et al.^24^ The input file for MESMER is given in the Supporting Information.

MESMER provides the potential to fit simulations for *k*_1_ to the observed values by varying the barrier height to decomposition, ⟨Δ*E*⟩_down_, and the imaginary frequency for the transition state. However, it was not possible to achieve a good unique fit to the data by varying all three parameters simultaneously. To fit to the data, a series of fits were performed in which the barrier height to decomposition was varied for a range of fixed values for ⟨Δ*E*⟩_down_ and the imaginary frequency. The best fit was determined by comparing the reduced χ^2^ statistic for the fits (shown in the Supporting Information), with the optimum fit using a value for ⟨Δ*E*⟩_down_ of 300 cm^–1^ and giving a barrier height of 67.2 ± 1.3 kJ mol^–1^ (compared to the calculated value^13^ of 70.3 kJ mol^–1^) using an imaginary frequency of 1480 ± 45 cm^–1^ (compared to the calculated value^13^ of 1619 cm^–1^).

Figure 5 shows the MESMER fit to the experimental data using the optimized values for ⟨Δ*E*⟩_down_, the barrier height, and the imaginary frequency. The impact of tunneling on the overall rate coefficients for *syn*-CH_3_CHOO decomposition is significant under atmospheric conditions. At 298 K and 760 Torr, the MESMER simulations indicate a rate coefficient of 150_–81_^+176^ s^–1^ when tunneling effects are included but only 5_–2_^+3^ s^–1^ when tunneling is not considered in the model. The MESMER simulations give a high pressure rate-limiting coefficient of 152_–83_^+183^ s^–1^ at 298 K.

Simulations were performed in MESMER to determine *k*_1_ using the optimized values for ⟨Δ*E*⟩_down_, the barrier height, and the imaginary frequency for (R1) at pressures between 1 and 7600 Torr and temperatures between 200 and 800 K. Simulations were also performed for *k*_2_ using the potential energy surface for *anti*-CH_3_CHOO isomerization (R2) provided by Vereecken et al.^13^ at the CCSD(T)/aug-cc-pVTZ//M06-ZX level of theory, with ⟨Δ*E*⟩_down_ = 300 cm^–1^ and the barrier height to isomerization adjusted by the same difference as that required for the fit to the experimental data for *syn*-CH_3_CHOO decomposition (i.e., using a barrier height of 62.6 kJ mol^–1^ for (R2) compared to the calculated value^13^ of 65.7 kJ mol^–1^). The difference in transition state structure for the unimolecular reactions of *syn*- and *anti*-CH_3_CHOO has a significant impact on the atmospheric chemistry of the two conformers, with quantum mechanical tunneling leading to decomposition being the major pathway for *syn*-CH_3_CHOO loss in the atmosphere but isomerization having limited importance for *anti*-CH_3_CHOO. Effects of tunneling were thus included in the simulations for (R1) but not for (R2), and the imaginary frequency for (R2) was not adjusted in the simulations compared to the calculated value reported by Vereecken et al.^13^ At 298 K and 760 Torr, the MESMER simulations indicate *k*_2_ = 54_–21_^+34^ s^–1^. The high-pressure rate-limiting coefficient is 64_–26_^+35^ s^–1^ at 298 K.

The rate coefficients calculated by MESMER using the optimized potential energy surface were parametrized using the Troe expression^46^ for use in kinetic models:
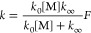
4The low- and high-pressure limiting rate coefficients, *k*_0_ and *k*_∞_ in eq 4, are given by eqs 5 and 6:
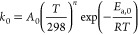
5
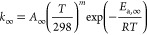
6The broadening factor, *F*, in eq 4 is given by eq 7:
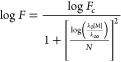
7where *N* is given by eq 8:
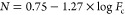
8The tunneling component to *k*_1_ was parametrized by the inclusion of an additional term given by
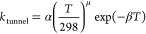
9Parametrization of *k*_1_ was thus achieved by the addition of eqs 4 and 9, while *k*_2_ was parametrized by eq 4, with terms in eq 4 defined by eqs 5–8. It should be noted that the variables provided by these parametrizations are not intended to provide physical insight into the reactions, which is provided by the Master equation calculations, but rather to provide descriptions of the kinetics that can be readily implemented in atmospheric models. Table 3 summarizes the fit parameters determined in eqs 4–9 to describe *k*_1_ and eqs 4–8 to describe *k*_2_. The MESMER simulations for *k*_1_ and *k*_2_ and fits to the eqs 4–9 (for *k*_1_) or eqs 4–8 (for *k*_2_) are shown in the Supporting Information.

**Table 3:** Summary of Fit Parameters to Describe MESMER Simulations for *k*_1_ (Eqs 4–9) and *k*_2_ (Eqs 4–8)

	*k*_1_/s^–1^	*k*_2_/s^–1^
*A*_0_/cm^3^ s^–1^	(1.9 ± 1.8) × 10^–4^	(7.1 ± 2.6) × 10^–4^
*n*	–8.08 ± 0.60	–7.78 ± 0.24
*E*_a,0_/kJ mol^–1^	63.5 ± 2.8	73.8 ± 1.0
*A*_∞_/s^–1^	(4.2 ± 4.0) × 10^9^	(1.0 ± 0.4) × 10^14^
*m*	3.10 ± 0.64	–2.64 ± 0.31
*E*_a,∞_/kJ mol^–1^	43.7 ± 2.6	69.7 ± 1.0
*F*_c_	0.729 ± 0.015	0.785 ± 0.015
α/s^–1^	801 ± 316	
μ	12.18 ± 0.46	
β/K^–1^	(8.8 ± 1.2) × 10^–3^	

### Atmospheric Implications

Table 4 gives the current IUPAC recommendations^20^ for the kinetics of stabilized *syn*- and *anti*-CH_3_CHOO with water vapor, water dimers, and SO_2_ at 298 K and 760 Torr, with typical concentrations for these species in the lower atmosphere and the resulting pseudo-first-order loss rates associated with each reaction.^20^ For *syn*-CH_3_CHOO, the pseudo-first-order loss for reaction with water vapor has an upper limit of 62 s^–1^, with the reaction of SO_2_ having a pseudo-first-order loss of 0.65 s^–1^. Decomposition is thus expected to dominate the atmospheric loss of stabilized *syn*-CH_3_CHOO, with the rate coefficient of 150 s^–1^ determined in this work for 298 K and 760 Torr, giving a lower limit of 70% for the loss of *syn*-CH_3_CHOO via decomposition for typical tropospheric conditions when considering the competition with water vapor and SO_2_. Quantum mechanical tunneling, which increases the rate coefficient for *syn*-CH_3_CHOO from 5_–2_^+3^ to 150_–81_^+176^ s^–1^ at 298 K and 760 Torr, thus dominates the atmospheric chemistry of *syn*-CH_3_CHOO, shifting the balance of the main atmospheric loss process from reaction with water to unimolecular decomposition. The production of OH radicals following the decomposition of *syn*-CH_3_CHOO, through the vinyl hydroperoxide mechanism, has potentially significant impacts on tropospheric oxidizing capacity at night and in low-light conditions when photolytic sources are OH are low.^2,3^ At low temperatures, the tunneling component to the decomposition of *syn*-CH_3_CHOO will dominate the fate of *syn*-CH_3_CHOO in the atmosphere; however, there are currently no experimental measurements of the kinetics of *syn*-CH_3_CHOO with water dimers which may increase atmospheric losses through channels other than decomposition at higher temperatures. For *anti*-CH_3_CHOO, atmospheric losses are expected to be dominated by reactions with water vapor and water dimers, for which the pseudo-first-order losses are orders of magnitude faster than isomerization. For both *syn*- and *anti*-CH_3_CHOO the products of reactions with water and water dimers are currently uncertain, limiting full assessment of the atmospheric impacts of these reactions.

**Table 4:** Current IUPAC Recommendations for Kinetics of *syn*- and *anti*-CH_3_CHOO with Water Vapor, Water Dimers, and SO_2_ at 298 K and 760 Torr,^20^ with Pseudo-First-Order Loss Rate Coefficients for Typical Atmospheric Concentrations Compared to the Kinetics for *syn*-CH_3_CHOO Decomposition and *anti*-CH_3_CHOO Isomerization determined at 298 K and 760 Torr in This Work^a^

reaction	conc^n^ of reaction partner/cm^–3^	*k*/cm^3^ s^–1^ or s^–1^	pseudo-first-order loss/s^–1^	% of total loss
*syn-*CH_3_CHOO + H_2_O	3.08 × 10^17^	<2 × 10^–16^	<62	<29.02
*syn*-CH_3_CHOO + (H_2_O)_2_	1.96 × 10^14^			
*syn*-CH_3_CHOO + SO_2_	2.50 × 10^10^	2.6 × 10^–11^	0.65	>0.31
*syn*-CH_3_CHOO decomposition		150	150	>70.67
*anti*-CH_3_CHOO + H_2_O	3.08 × 10^17^	1.3 × 10^–14^	4000	31.56
*anti*-CH_3_CHOO + (H_2_O)_2_	1.96 × 10^14^	4.4 × 10^–11^	8600	67.98
*anti*-CH_3_CHOO + SO_2_	2.50 × 10^10^	1.4 × 10^–10^	3.5	0.03
*anti*-CH_3_CHOO isomerization		54	54	0.43

a Concentrations of reaction partners used are those adopted by IUPAC for the lower troposphere.^20^

## Conclusions

The kinetics of the unimolecular decomposition of the stabilized Criegee intermediate *syn*-CH_3_CHOO (*k*_1_) have been investigated at temperatures between 297 and 331 K and pressures between 12 and 300 Torr using laser flash photolysis of CH_3_CHI_2_/O_2_/N_2_ gas mixtures coupled with time-resolved broadband UV absorption spectroscopy.

Master equation fits to experimental results for *k*_1_ were performed using MESMER. The fits required a decrease in the calculated^13^ barrier height of 70.3 to 67.2 kJ mol^–1^ and in the calculated^13^ imaginary frequency for the reaction from a value of 1619 to 1480 cm^–1^, using an exponential down model to describe collisional energy transfer with ⟨Δ*E*⟩_down_ = 300 cm^–1^. The effects of quantum mechanical tunneling were included in MESMER using the asymmetric Eckart tunneling model, indicating significant impacts under atmospheric conditions. MESMER simulations using the optimized barrier height and imaginary frequency indicate a rate coefficient for decomposition of stabilized *syn*-CH_3_CHOO of 150_–81_^+176^ s^–1^ when tunneling effects are included but only 5_–2_^+3^ s^–1^ when the effects of tunneling are not considered.

MESMER simulations were also performed for the unimolecular isomerization of the stabilized Criegee intermediate *anti*-CH_3_CHOO using a calculated^13^ barrier height adjusted by the same difference required to fit the experimental results for *syn*-CH_3_CHOO, indicating a rate coefficient of 54_–21_^+34^ s^–1^ at 298 K and 760 Torr.

Under atmospheric conditions, the impact of tunneling is expected to make decomposition the dominant loss mechanism for stabilized *syn*-CH_3_CHOO, while the isomerization of stabilized *anti*-CH_3_CHOO is expected to be a minor loss process.

## Supporting Information Available

Description of the instrument response function; comparison of first- and mixed-order fits; temperature dependence of *syn*-CH_3_CHOO + CH_3_CHI_2_; physical losses of *syn*-CH_3_CHOO; optimization of the potential energy surface for (R1) using MESMER; MESMER analysis of *k*_1_ and *k*_2_; MESMER input files (PDF)
