# Supplementary material for: Unimolecular Kinetics of Stabilized CH3CHOO Criegee Intermediates: syn-CH3CHOO Decomposition and anti-CH3CHOO Isomerization
Source: J Phys Chem A. 2022 Sep 23;126(39):6984–94. doi: 10.1021/acs.jpca.2c05461 (PMC9549458; doi:10.1021/acs.jpca.2c05461)
Supplement: Supplementary file 1 — jp2c05461_si_001.pdf [file jp2c05461_si_001.pdf]

Unimolecular Kinetics of Stabilised CH_3CHOO Criegee Intermediates: *syn*-CH_3CHOO Decomposition and *anti*-CH_3CHOO Isomerisation

Callum Robinson,[1] Lavinia Onel,[1] James Newman,[1] Rachel Lade,[1] Kendrew Au,[2] Leonid Sheps,[2] Dwayne E. Heard,[1] Paul W. Seakins,[1] Mark A. Blitz[1,3] and Daniel Stone[1*]

[1] School of Chemistry, University of Leeds, Woodhouse Lane, Leeds, LS2 9JT, UK

[2] Combustion Research Facility, Sandia National Laboratories, Livermore, CA 94551, USA

[3] National Centre for Atmospheric Science, School of Chemistry, University of Leeds, Woodhouse Lane, Leeds, LS2 9JT, UK

*Corresponding author: Daniel Stone d.stone@leeds.ac.uk

Supporting Information

Table of contents

Instrument response function p S2

Comparison of mixed-order and first-order fits p S3

Temperature dependence of *syn*-CH_3CHOO + CH_3CHI_2 pS7

Physical losses of *syn*-CH_3CHOO pS8

Optimisation of the potential energy surface for R1 using MESMER pS9

MESMER analysis of k_1 and k_2 pS10

References pS11

MESMER input file for *syn*-CH_3CHOO pS12

MESMER input file for *anti*-CH_3CHOO pS23

Instrument response function

Experiments at T > 297 K were performed using the integrated spectrograph and thermoelectrically cooled charge-coupled device (CCD) detector (FER-SCI-1024BRX, Princeton Instruments) to provide increased time resolution. Concentration-time profiles for these experiments are given by a convolution of the 'true' kinetic decay with an instrument response function (IRF) which results from the simultaneous illumination of multiple rows on the CCD and the row-by-row shifting of photocharge from the illuminated region of the CCD to a storage region. The IRF can be described by a Gaussian function with peak height a centred at t_c and with width w (Equation S1):

$$f(t) = a\ exp\left(-\frac{(t-t_c)^2}{2w^2}\right)$$

(Equation S1)

The 'true' kinetic profiles for *syn*-CH_3CHOO are given by Equations S2 (mixed first- and second-order) or S3 (first-order).

$$C_t = \frac{C_0 k'}{k'\ exp(k't) - 2k''C_0 + 2k''C_0\ exp\ (k't)}$$

(Equation S2)

where C_t is the concentration of *syn*-CH_3CHOO at time t, C_0 is the initial concentration of *syn*-CH_3CHOO, k' represents the first-order (or pseudo-first-order) losses of *syn*-CH_3CHOO and k'' represents the second-order losses of *syn*-CH_3CHOO.

$$C_t = \ C_0\ exp(-k't)$$

(Equation S3)

where C_t is the concentration of *syn*-CH_3CHOO at time t, C_0 is the initial concentration of *syn*-CH_3CHOO, and k' is the pseudo-first-order rate coefficient describing the loss of *syn*-CH_3CHOO.

Convolution of Equations S2 and S3 with the IRF gives Equations S4 and S5, respectively.

$$C_t = \left\{\frac{1}{\left(\frac{1}{C_0} + \frac{2k''}{k'}\right)}\right\} \exp\left\{\frac{(k'w)^2}{2} - k'\,(t - \ t_c) + \frac{2k''}{k'}\right\} \times \frac{\left\{1 + \text{erf}\left(\frac{t - t_c - k'w^2}{\sqrt{2}\,w}\right)\right\}}{2}$$

(Equation S4)

$$C_t = \frac{C_0}{2}\ exp\left\{\frac{(k'w)^2}{2} - k'(t - t_c)\right\} \times \left\{1 + \text{erf}\left(\frac{t -\ t_c - k'w^2}{\sqrt{2}w}\right)\right\}$$

(Equation S5)

where erf is the error function obtained in the integration of the normalised form of the Gaussian function.

Equations S4 and S5 were fit to the observed concentration-time profiles for *syn*-CH_3CHOO (see discussion below), with the IRF parameters t_c and w treated as global parameters.

Comparison of mixed-order and first-order fits

Concentration-time profiles for *syn*-CH_3CHOO were fit to both a mixed first- and second-order kinetic equation (Equation S2, coupled with the IRF for $T > 297$ K) and to a first-order kinetic equation (Equation S3, coupled with the IRF for $T > 297$ K) to investigate the potential impacts of any second-order reactions such as *syn*-CH_3CHOO + *syn*-CH_3CHOO or *syn*-CH_3CHOO + I. For fits to the mixed-order equation, the second-order component (k'' in Equations S2 and S4) was treated as a global parameter at each temperature and pressure.

Figure S1 shows a comparison between the mixed-order and first-order fits to a typical concentration-time profile for *syn*-CH_3CHOO at $T = 297$ K, which indicates a poorer fit quality for the first-order fit compared to the mixed-order fit. Figure S2 shows the comparisons between the decomposition rate coefficients for *syn*-CH_3CHOO obtained from the mixed-order fits and those obtained from the first-order fits.

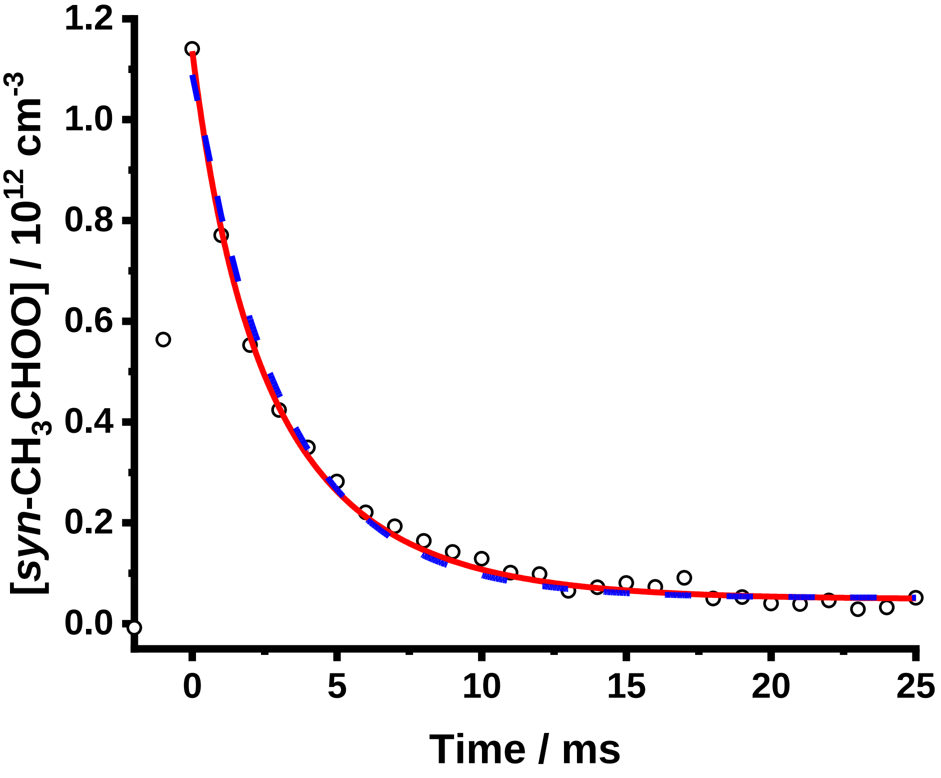

Figure S1: Comparison of fits to observed *syn*-CH_3CHOO data (black) obtained with the mixed-order kinetic analysis (Equation S2) (solid red line) and the first-order kinetic analysis (Equation S3) (dashed blue line). For these data, $T = 297$ K, $p = 12$ Torr, and $[CH_3CHI_2] = 2.6 \times 10^{13}$ cm^{-3}. The fit to Equation S2 gave [*syn*-$CH_3CHOO]_0$ = $(1.09 \pm 0.01) \times 10^{12}$ cm^{-3}, $k' = (237 \pm 10)$ s^{-1} , and $k'' = (8.65 \pm 0.49) \times 10^{-11}$ cm^3 s^{-1} , while that to Equation S3 gave [*syn*-$CH_3CHOO]_0 = (1.04 \pm 0.02) \times 10^{12}$ cm^{-3} and $k' = (315 \pm 10)$ s^{-1}. Errors are 1σ.

a)

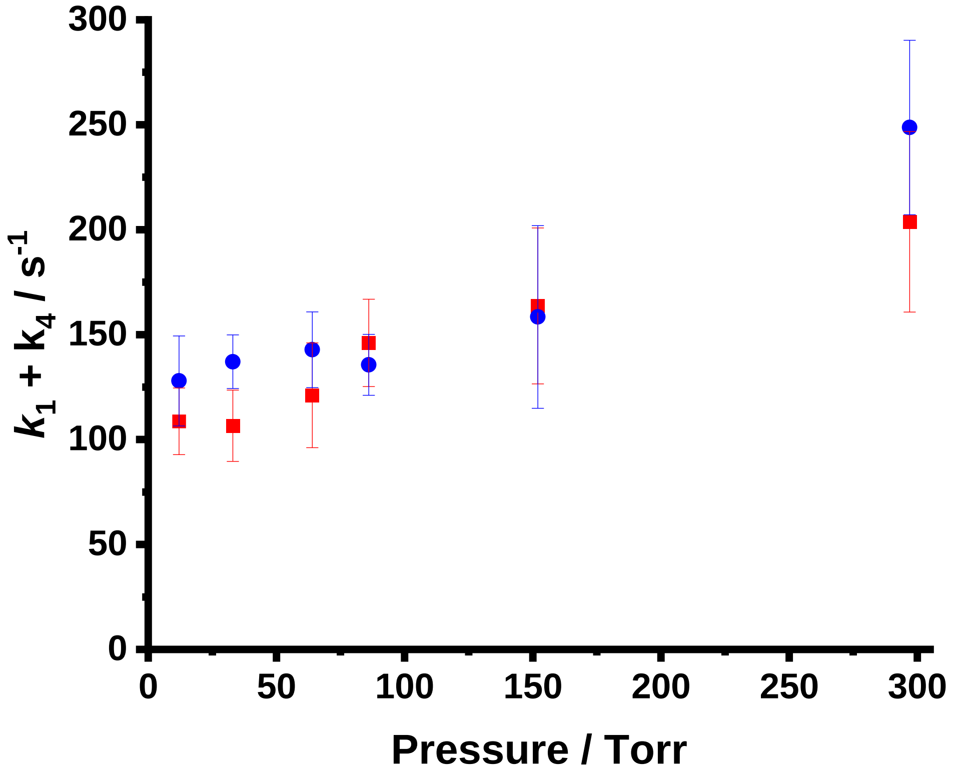
300
250
200
150
100
50
0
0
50
100
150
200
250
300
k_1 + k$_4$ / s^{-1}
Pressure / Torr

b)

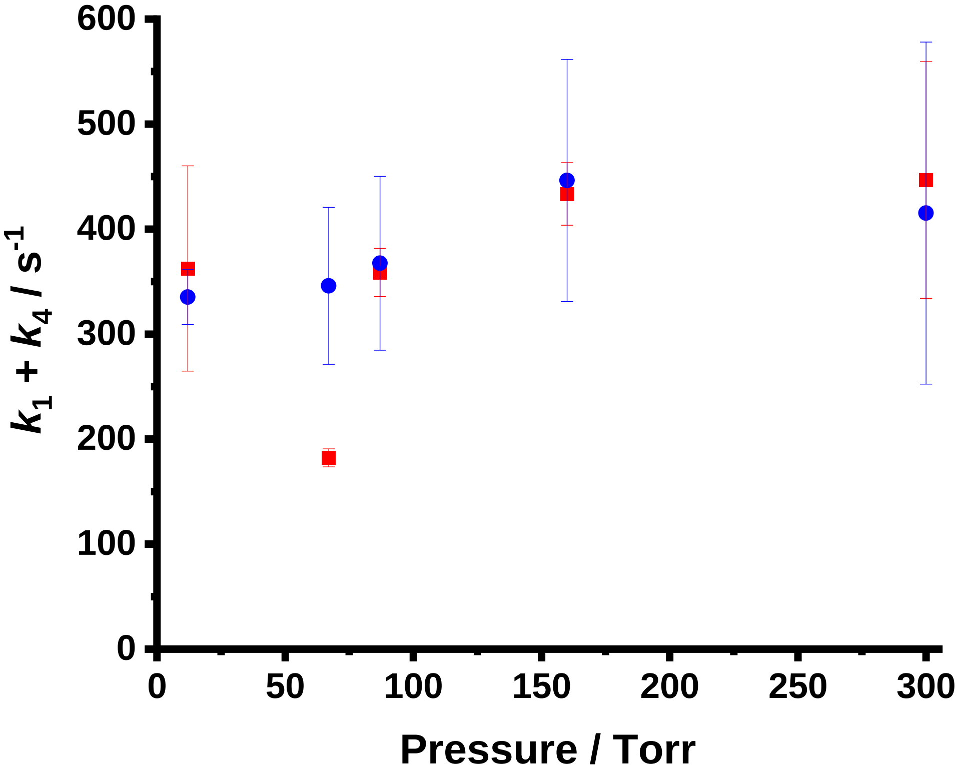
600
500
400
300
200
100
0
0
50
100
150
200
250
300
$k_1 + k_4$ / s^{-1}
Pressure / Torr

c)

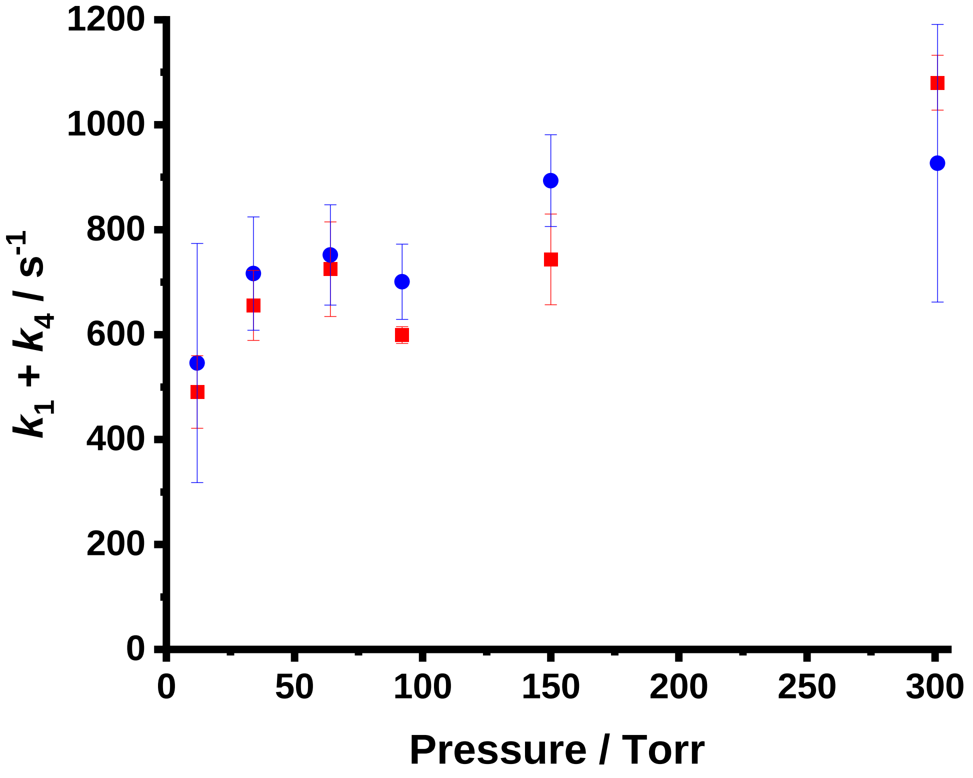

Figure S2: Comparison of results for k_1 + k_4 obtained from fits to the mixed-order kinetic analysis (using Equations S2 (T = 297 K) and S4 (T > 297 K)) (red) and the first-order kinetic analysis (using Equations S3 (T = 297 K) and S5 (T > 297 K)) (blue) for data at a) T = 297 K; b) T = 314 K; and c) T = 331 K.

For experiments at T = 297 K the fits to the mixed-order analysis were sensitive to the second-order component, giving an average value of (7.8 ± 3.0) × 10^{-11} cm^3 s^{-1}, which did not display any significant dependence on pressure (Figure S3), and the first-order components were typically lower than those obtained from the first-order fits.

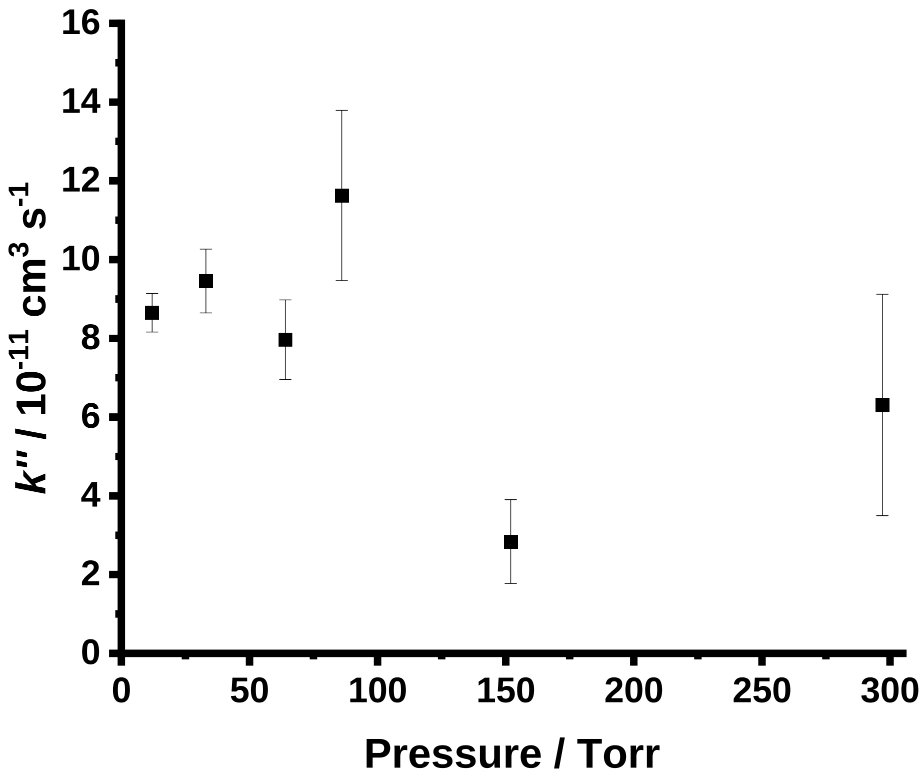

Figure S3: Values for *k''* determined at $T = 297$ K as a function of pressure.

For data at $T > 297$ K, fits to the mixed-order equation were insensitive to the second-order component, and there was no significant difference between the fit quality or fit results for the first-order component to the loss of *syn*-CH_3CHOO obtained from the mixed-order or first-order equations.

Thus, at $T = 297$ K it is likely that there is a significant contribution of second-order processes such as *syn*-CH_3CHOO + *syn*-CH_3CHOO or *syn*-CH_3CHOO + I to the observed *syn*-CH_3CHOO, but such processes are not significant at $T > 297$ K owing to likely negative temperature dependences of *syn*-CH_3CHOO + *syn*-CH_3CHOO and *syn*-CH_3CHOO + I in competition with the positive temperature dependences of *syn*-CH_3CHOO + CH_3CHI_2 and *syn*-CH_3CHOO decomposition.

Data reported in this work for the kinetics of *syn*-CH_3CHOO at $T = 297$ K were obtained from the mixed-order fits while data at $T > 297$ K were obtained from the first-order fits.

Temperature dependence of *syn*-CH_3CHOO + CH_3CHI_2

The kinetics of the reaction between *syn*-CH_3CHOO + CH_3CHI_2 (R3, main text) were obtained from plots of the k' against [CH_3CHI_2] (as described in the main text and shown in Figure 4, main text). At 297 K, a value of k_3 = (5.1 ± 2.4) × 10^{-12} cm^3 s^{-1} was obtained, while results at 314 K gave k_3 = (6.7 ± 4.5) × 10^{-12} cm^3 s^{-1} and those at 351 K gave k_3 = (7.9 ± 1.9) × 10^{-12} cm^3 s^{-1}. Figure S4 shows the temperature dependence of k_3, which can be described by k_3 = (3.2 ± 0.7) × 10^{-10} exp((–1230±70)/T) cm^3 s^{-1}.

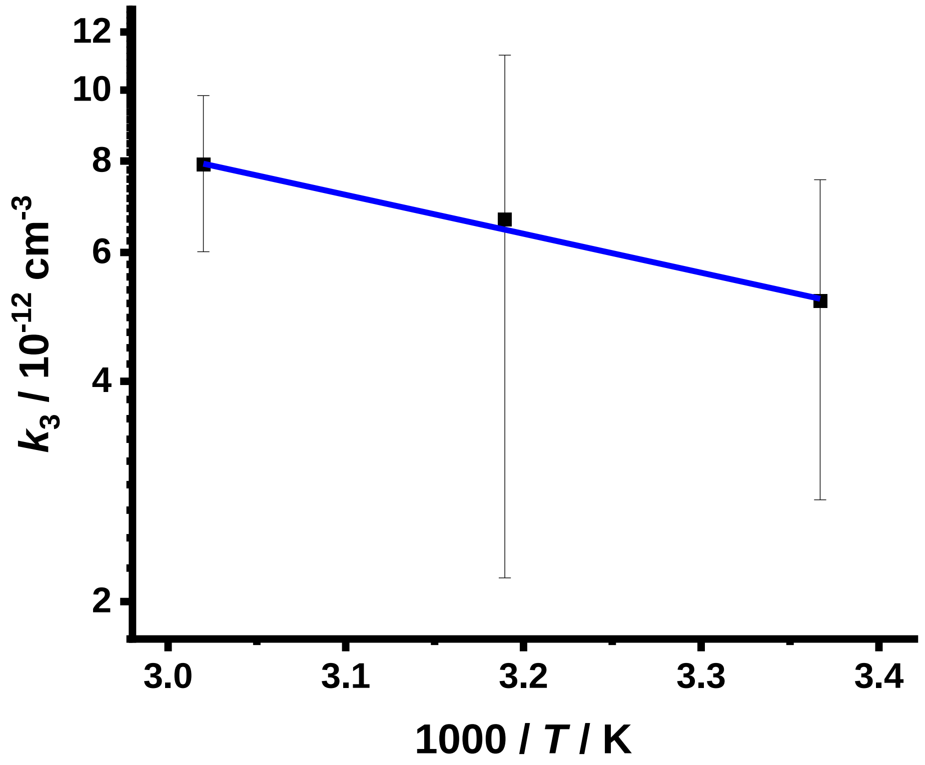

Figure S4: Temperature dependence of k_3. The fit to the data (solid blue line) can be described by k_3 = (3.2 ± 0.7) × 10^{-10} exp((–1230±70)/T) cm^3 s^{-1}.

Physical losses of *syn*-CH_3CHOO

Effects of physical losses were estimated from experiments in which the physical losses of the Criegee intermediate CH_2OO were measured in experiments involving photolysis of $CH_2I_2/O_2/N_2$ mixtures,[1] in which losses of CH_2OO result from reactions SR1-SR4:

$CH_2OO + CH_2OO \rightarrow 2\ HCHO + O_2$ (SR1)

$CH_2OO + I \rightarrow$ products (SR2)

$CH_2OO \rightarrow$ decomposition (SR3)

$CH_2OO \rightarrow$ physical losses (SR4)

Our previous work[2] has demonstrated that the decomposition kinetics of CH_2OO are slow ($k_{SR3} << 1\ s^{-1}$) at 298 K and thus first-order losses of CH_2OO are dominated by physical losses (SR4). Rate coefficients describing the contribution of physical losses to CH_2OO (k_{SR4}) were determined for our experimental setup as described in our previous work[1] and are shown in Figure S5. For pressures above 12 Torr, k_{SR4} shows no significant dependence on pressure, with a mean value of $(3.2 \pm 1.7)\ s^{-1}$, while at a pressure of 12 Torr a value of $(10.6 \pm 5.9)\ s^{-1}$ was obtained. A similar value of $(9 \pm 6)\ s^{-1}$ was reported by Li *et al.*[3] for a similar experimental setup, with no significant dependence on temperature or pressure.

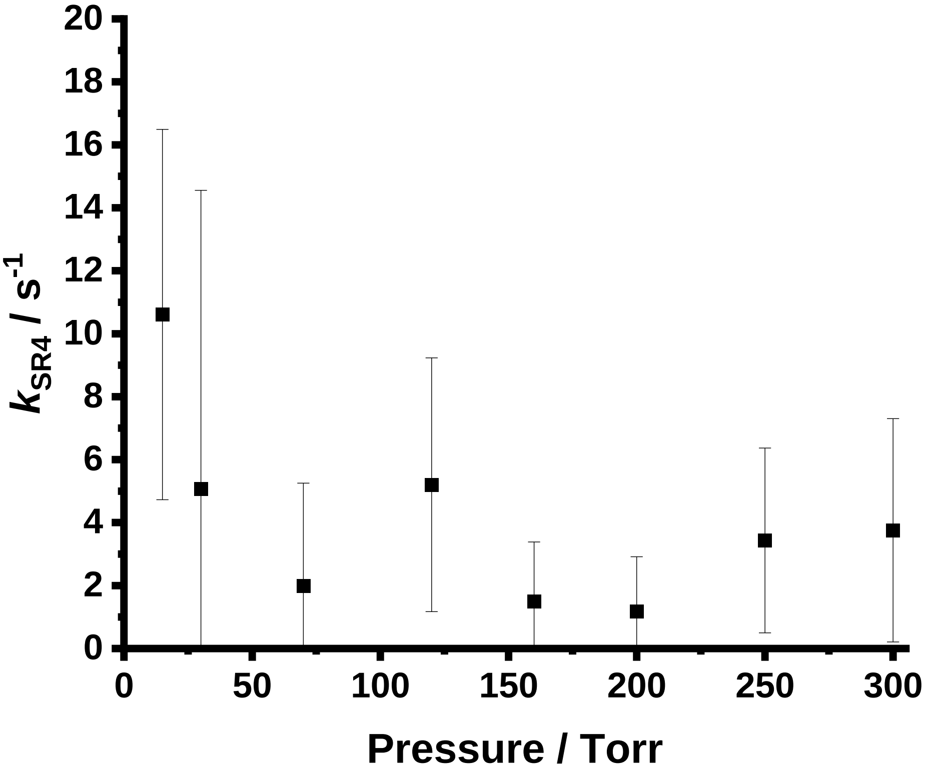

Figure S5: First-order rate coefficients used to describe the physical losses of CH_2OO (k_{SR4}) in experiments involving photolysis of $CH_2I_2/O_2/N_2$ mixtures[1] at room temperature used to estimate the diffusion kinetics of *syn*-CH_3CHOO (k_4).

Optimisation of the potential energy surface for R1 using MESMER

The potential energy surface describing R1 was fit to the experimental results by varying the barrier height to decomposition, $\langle \Delta E \rangle_{down}$, and the imaginary frequency for the transition state in MESMER and comparing the observed rate coefficients (k_1) with those calculated by MESMER. While MESMER does provide the potential to fit all three parameters to experimental data, it was not possible to achieve a good unique fit to the data by varying all three parameters simultaneously in this work. In order to fit to the data, a series of fits were performed in which the barrier height to decomposition was varied for a range of fixed values for $\langle \Delta E \rangle_{down}$ and the imaginary frequency. The best fit was determined by comparing the reduced χ^2 statistic for the fits, with the optimum fit giving a value for $\langle \Delta E \rangle_{down}$ of 300 cm^{-1}, a barrier height of 67.2 kJ mol^{-1} (compared to the calculated value[4] of 70.3 kJ mol^{-1}), and an imaginary frequency of 1480 cm^{-1} (compared to the calculated value[4] of 1619 cm^{-1}). Figure S6 shows the variation in the reduced χ^2 statistic as a function of $\langle \Delta E \rangle_{down}$, the barrier height to decomposition, and the imaginary frequency.

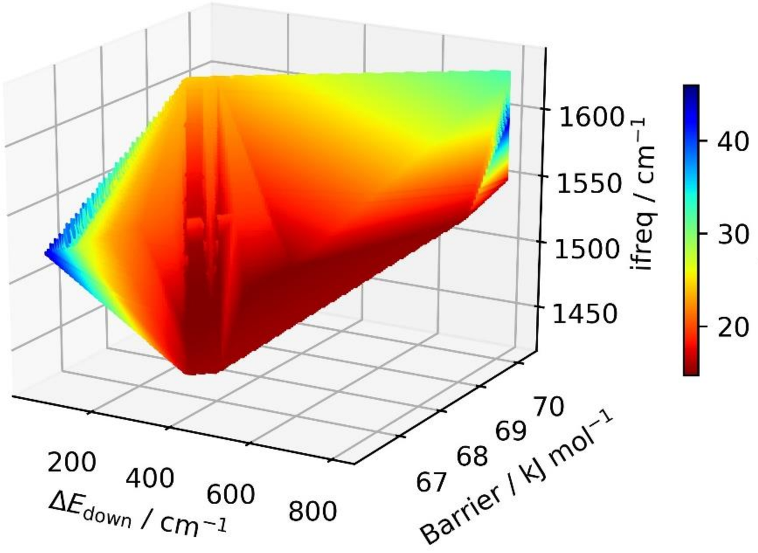

Figure S6: Variation in the reduced χ^2 statistic used to compare observed values of k_1 with calculated values in MESMER as a function of $\langle \Delta E \rangle_{down}$, the barrier height to decomposition, and the imaginary frequency.

MESMER analysis of k_1 and k_2

MESMER simulations were performed using the potential energy surface reported by Vereecken *et al.*[4] with the barrier height determined in this work (67.2 kJ mol^{-1}), $\langle \Delta E \rangle_{down}$ = 300 cm^{-1}, and an imaginary frequency of 1480 cm^{-1} to calculate *syn*-CH_3CHOO decomposition kinetics (k_1) at temperatures between 200 and 800 K and pressures between 1 and 7600 Torr. The output was parameterised using a Troe expression coupled with an expression to parameterise the effects of tunnelling, as detailed in the main text.[5] Figure S7 shows the fits to the MESMER output for k_1 to provide the parameterisation.

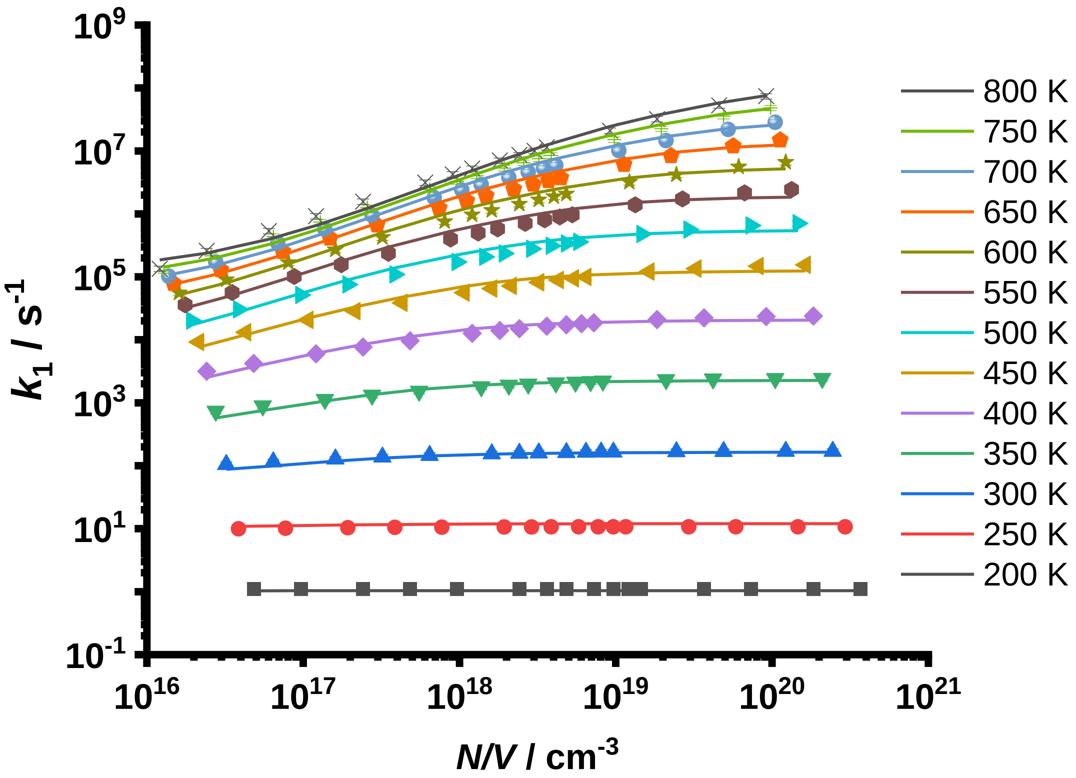

Figure S7: Fits to using Equations 4-9 in the main text (solid lines) to MESMER simulations for k_1 (filled data points) using a barrier height of 67.2 kJ mol^{-1}, $\langle \Delta E \rangle_{down}$ = 300 cm^{-1}, and an imaginary frequency of 1480 cm^{-1}.

Simulations were also performed to determine k_2 using the potential energy surface reported by Vereecken *et al*. with $\langle \Delta E \rangle_{down}$ = 300 cm^{-1} and the barrier height adjusted by the same difference as that required for the fit to the experimental data for *syn*-CH_3CHOO (i.e. using a barrier height of 62.6 kJ mol^{-1} for R2 compared to the calculated value[4] of 65.7 kJ mol^{-1}. The output was parameterised using a Troe expression, as detailed in the main text.[5] Figure S8 shows the fits to the MESMER output for k_2 to provide the parameterisation.

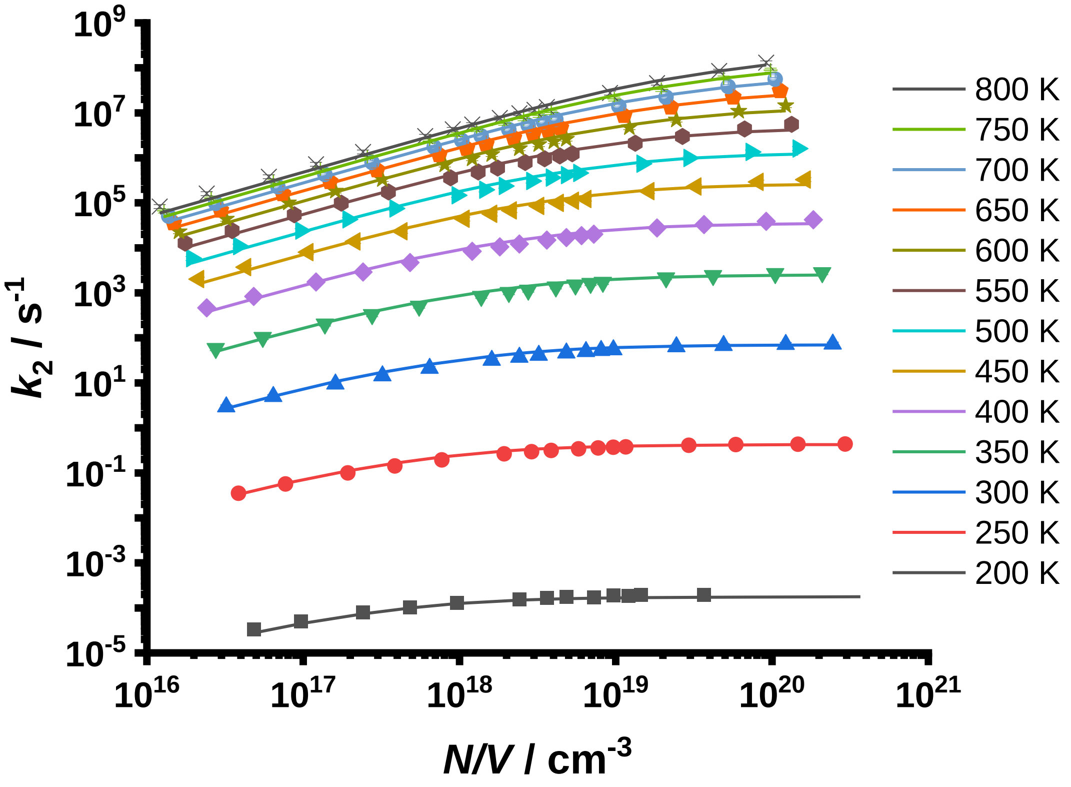

Figure S8: Fits using Equations 4-8 in the main text (solid lines) to MESMER simulations for k_2 (filled data points) using a barrier height of 62.6 kJ mol^{-1} and $\langle \Delta E \rangle_{down}$ = 300 cm^{-1}.

References

1. Mir, Z. S.; Lewis, T. R.; Onel, L.; Blitz, M. A.; Seakins, P. W.; Stone, D., CH_2OO Criegee Intermediate UV Absorption Cross-Sections and Kinetics of CH_2OO + CH_2OO and CH_2OO + I as a Function of Pressure. *Phys. Chem. Chem. Phys.* **2020,** *22*, 9448-9459.

2. Stone, D.; Au, K.; Sime, S.; Medeiros, D. J.; Blitz, M.; Seakins, P. W.; Decker, Z.; Sheps, L., Unimolecular Decomposition Kinetics of the Stabilised Criegee Intermediates CH_2OO and CD_2OO. *Phys. Chem. Chem. Phys.* **2018,** *20*, 24940-24954.

3. Li, Y. L.; Kuo, M. T.; Lin, J. M., Unimolecular Decomposition Rates of a Methyl-Substituted Criegee Intermediate *syn*-CH_3CHOO. *RSC Advances* **2020,** *10*, 8518-8524.

4. Vereecken, L.; Novelli, A.; Taraborrelli, D., Unimolecular Decay Strongly Limits the Atmospheric Impact of Criegee Intermediates. *Phys. Chem. Chem. Phys.* **2017,** *19*, 31599-31612.

5. Troe, J., Predictive Possibilities of Unimolecular Rate Theory. *J. Phys. Chem.* **1979,** *83*, 114-126.

MESMER input file for *syn*-CH_3CHOO


```
<?xml version="1.0" encoding="utf-8" ?>

<?xml-stylesheet type='text/xsl' href='../../mesmer2.xsl' media='other'?>

<?xml-stylesheet type='text/xsl' href='../../mesmer1.xsl' media='screen'?>

<me:mesmer xmlns="http://www.xml-cml.org/schema" xmlns:me="http://www.chem.leeds.ac.uk/mesmer"
xmlns:xsi="http://www.w3.org/2001/XMLSchema-instance" xmlns:cml="http://www.xml-cml.org/schema">


<me:title>syn C2 decomposition</me:title>

<moleculeList>

 <molecule id="synC2">

  <atomArray>

   <atom id="a1" elementType="C" spinMultiplicity="2" x3="0.481451" y3="0.698499" z3="0.000003"/>

   <atom id="a2" elementType="H" x3="0.813825" y3="1.729288" z3="-0.000011"/>

   <atom id="a3" elementType="C" x3="1.364688" y3="-0.472562" z3="-0.000005"/>

   <atom id="a4" elementType="H" x3="2.410743" y3="-0.180623" z3="-0.000177"/>

   <atom id="a5" elementType="H" x3="1.131036" y3="-1.097642" z3="0.867144"/>

   <atom id="a6" elementType="H" x3="1.130756" y3="-1.097813" z3="-0.866950"/>

   <atom id="a7" elementType="O" x3="-0.775497" y3="0.588342" z3="0.000003"/>

   <atom id="a8" elementType="O" spinMultiplicity="2" x3="-1.294901" y3="-0.676946" z3="-0.000002"/>

  </atomArray>

  <bondArray>

   <bond atomRefs2="a6 a3" order="1"/>
```

<bond atomRefs2="a4 a3" order="1"/>
<bond atomRefs2="a2 a1" order="1"/>
<bond atomRefs2="a3 a1" order="1"/>
<bond atomRefs2="a3 a5" order="1"/>
<bond atomRefs2="a8 a7" order="1"/>
<bond atomRefs2="a1 a7" order="1"/>
</bondArray>
<propertyList>
<property dictRef="me:ZPE">
<scalar units="kJ/mol">0</scalar>
</property>
<property dictRef="me:rotConsts">
<array units="cm-1">0.59538 0.241943 0.177514</array>
</property>
<property dictRef="me:vibFreqs">
<array units="cm-1">216.1377 321.0560 471.8577 683.1234 781.1765 928.5602 998.9468 1068.7093 1112.1445 1361.4043 1410.3058 1440.5155 1464.6823 1647.0242 3053.7590 3098.7604 3190.5974 3206.81609</array>
</property>
<property dictRef="me:frequenciesScaleFactor">
<scalar>1</scalar>
</property>
<property dictRef="me:symmetryNumber">
<scalar>1</scalar>

</property>
<property dictRef="me:MW">
<scalar units="amu">60</scalar>
</property>
<property dictRef="me:spinMultiplicity">
<scalar>3</scalar>
</property>
</propertyList>
<me:DOSCMethod name="ClassicalRotors"/>
<me:energyTransferModel xsi:type="me:ExponentialDown">
<me:deltaEDown units="cm-1">300</me:deltaEDown>
</me:energyTransferModel>
</molecule>

<molecule id="TS">
<atomArray>
<atom id="a1" elementType="C" spinMultiplicity="2" x3="0.559265" y3="0.705585" z3="0.035556"/>
<atom id="a2" elementType="H" x3="0.950560" y3="1.705795" z3="-0.107141"/>
<atom id="a3" elementType="C" x3="1.233516" y3="-0.519592" z3="-0.016433"/>
<atom id="a4" elementType="H" x3="2.254536" y3="-0.492744" z3="-0.372924"/>
<atom id="a5" elementType="H" x3="1.087498" y3="-1.189249" z3="0.830886"/>
<atom id="a6" elementType="H" x3="0.086616" y3="-1.037481" z3="-0.474838"/>

<atom id="a7" elementType="O" x3="-0.736491" y3="0.665960" z3="0.027798"/>
<atom id="a8" elementType="O" spinMultiplicity="2" x3="-1.155497" y3="-0.678745" z3="-0.026638"/>
</atomArray>
<bondArray>
<bond atomRefs2="a6 a3" order="1"/>
<bond atomRefs2="a4 a3" order="1"/>
<bond atomRefs2="a2 a1" order="1"/>
<bond atomRefs2="a8 a7" order="1"/>
<bond atomRefs2="a3 a1" order="1"/>
<bond atomRefs2="a3 a5" order="1"/>
<bond atomRefs2="a7 a1" order="1"/>
</bondArray>
<propertyList>
<property dictRef="me:ZPE">
<scalar units="kJ/mol">67.1676</scalar>
</property>
<property dictRef="me:rotConsts">
<array units="cm-1"> 0.564090748 0.283814931 0.19367493 </array>
</property>
<property dictRef="me:vibFreqs">
<array units="cm-1"> 503.4335 534.7613 713.5631 756.1878 870.5213 968.7228 989.7768 1056.4046 1217.3921 1323.4907 1359.5181 1493.5595 1593.7212 1880.7527 3111.4342 3205.1557 3239.0718</array>
</property>

<property title="ImaginaryFrequency" dictRef="me:imFreqs">
<array units="cm-1"> 1480 </array>
</property>
<property dictRef="me:frequenciesScaleFactor">
<scalar>1</scalar>
</property>
<property dictRef="me:symmetryNumber">
<scalar>1</scalar>
</property>
<property dictRef="me:MW">
<scalar units="amu">60</scalar>
</property>
<property dictRef="me:spinMultiplicity">
<scalar>3</scalar>
</property>
</propertyList>
<me:DOSCMethod name="ClassicalRotors"/>
</molecule>

<molecule id="VHP">
<atomArray>
<atom id="a1" elementType="C" x3="-0.711005" y3="0.555783" z3="0.012967"/>

<atom id="a2" elementType="H" x3="-1.141679" y3="1.549082" z3="0.034816"/>
<atom id="a3" elementType="H" x3="-2.497705" y3="-0.487454" z3="0.033399"/>
<atom id="a4" elementType="C" x3="-1.422271" y3="-0.561487" z3="0.008612"/>
<atom id="a5" elementType="H" x3="-0.965840" y3="-1.536119" z3="-0.030796"/>
<atom id="a6" elementType="O" x3="0.647526" y3="0.710445" z3="-0.018173"/>
<atom id="a7" elementType="O" x3="1.307633" y3="-0.573770" z3="-0.097132"/>
<atom id="a8" elementType="H" x3="1.763602" y3="-0.584683" z3="0.755546"/>
</atomArray>
<bondArray>
<bond atomRefs2="a7 a6" order="1"/>
<bond atomRefs2="a7 a8" order="1"/>
<bond atomRefs2="a5 a4" order="1"/>
<bond atomRefs2="a6 a1" order="1"/>
<bond atomRefs2="a4 a1" order="2"/>
<bond atomRefs2="a4 a3" order="1"/>
<bond atomRefs2="a1 a2" order="1"/>
</bondArray>
<propertyList>
<property dictRef="me:ZPE">
<scalar units="kJ/mol"> -76.5 </scalar>
</property>
<property dictRef="me:vibFreqs">

<array units="cm-1">169.8726 239.9509 330.9122 611.5948 706.5401 846.6469 864.9339 967.4421 969.7083 1156.37 1316.3823 1382.7587 1425.959 1695.6329 3188.197 3210.2282 3292.9973 3779.6216 </array>

</property>

<property dictRef="me:frequenciesScaleFactor">

<scalar>1</scalar>

</property>

<property dictRef="me:symmetryNumber">

<scalar>1</scalar>

</property>

<property dictRef="me:MW">

<scalar units="amu">60</scalar>

</property>

<property dictRef="me:spinMultiplicity">

<scalar>1</scalar>

</property>

</propertyList>

<me:DOSCMethod name="ClassicalRotors"/>

<me:energyTransferModel xsi:type="me:ExponentialDown">

<me:deltaEDown units="cm-1" derivedFrom="synC2:deltaEDown" factor="1.0" addand="0.0">300</me:deltaEDown>

</me:energyTransferModel>

</molecule>

<molecule id="He">

```
 <atom elementType="He" />
 <propertyList>
  <property dictRef="me:epsilon">
   <scalar>10.2</scalar>
  </property>
  <property dictRef="me:sigma">
   <scalar>2.55</scalar>
  </property>
  <property dictRef="me:MW">
   <scalar units="amu">4.0</scalar>
  </property>
 </propertyList>
</molecule>

<molecule id="N2">
 <atom elementType="N"/>
 <propertyList>
  <property dictRef="me:epsilon">
   <scalar>423.0</scalar>
  </property>
  <property dictRef="me:sigma">
   <scalar>5.39</scalar>
```

```
  </property>
  <property dictRef="me:MW">
   <scalar units="amu">28.0</scalar>
  </property>
 </propertyList>
</molecule>

</moleculeList>

<reactionList>
 <reaction id="R1">
  <reactant>
   <molecule ref="synC2" me:type="modelled" />
  </reactant>
  <product>
   <molecule ref="VHP" me:type="modelled" />
  </product>
  <me:MCRCMethod name="SimpleRRKM"/>
  <me:transitionState>
   <molecule ref="TS" me:type="transitionState" />
  </me:transitionState>
 <me:tunneling xsi:type="Eckart">
```

```
 </me:tunneling>
 </reaction>
</reactionList>

<me:conditions>
 <me:bathGas>N2</me:bathGas>
 <me:PTs>

 <me:PTpair me:units="Torr" me:P="760" me:T="298" me:precision= "d" />

 </me:PTs>
</me:conditions>

<me:modelParameters>
 <me:grainSize units="cm-1">50</me:grainSize>
 <me:energyAboveTheTopHill>25.0</me:energyAboveTheTopHill>
</me:modelParameters>

<me:control>
 <me:testDOS />
 <me:printSpeciesProfile />
 <me:testRateConstant />
```

<me:printGrainDOS />
<me:printGrainkfE />
<me:printGrainkbE />
<me:eigenvalues>0</me:eigenvalues>
</me:control>

</me:mesmer>

MESMER input file for *anti*-CH_3CHOO


```
<?xml version="1.0" encoding="utf-8" ?>
<?xml-stylesheet type='text/xsl' href='../../mesmer2.xsl' media='other'?>
<?xml-stylesheet type='text/xsl' href='../../mesmer1.xsl' media='screen'?>
<me:mesmer xmlns="http://www.xml-cml.org/schema" xmlns:me="http://www.chem.leeds.ac.uk/mesmer"
xmlns:xsi="http://www.w3.org/2001/XMLSchema-instance" xmlns:cml="http://www.xml-cml.org/schema">

<me:title>anti C2 decomposition</me:title>
<moleculeList>
 <molecule id="antiC2">
 <propertyList>
  <property dictRef="me:ZPE">
   <scalar units="kJ/mol">0</scalar>
  </property>
  <property dictRef="me:rotConsts">
   <array units="cm-1"> 1.674024624 0.149440341 0.140753668 </array>
  </property>
  <property dictRef="me:vibFreqs">
   <array units="cm-1"> 167.653 268.3336 329.1509 566.4967 883.501 901.6039 986.5165 1094.5197 1168.611 1362.482 1421.7142 1467.7291 1469.4722
1672.6658 3064.4357 3120.6224 3166.0899 3183.3546 </array>
  </property>
  <property dictRef="me:frequenciesScaleFactor">
   <scalar>1</scalar>
```

</property>
<property dictRef="me:symmetryNumber">
<scalar>1</scalar>
</property>
<property dictRef="me:MW">
<scalar units="amu">60</scalar>
</property>
<property dictRef="me:spinMultiplicity">
<scalar>3</scalar>
</property>
</propertyList>
<me:DOSCMethod name="ClassicalRotors"/>
<me:energyTransferModel xsi:type="me:ExponentialDown">
<me:deltaEDown units="cm-1">300</me:deltaEDown>
</me:energyTransferModel>
</molecule>

<molecule id="TS">
<propertyList>
<property dictRef="me:ZPE">
<scalar units="kJ/mol">62.5652</scalar>
</property>

<property dictRef="me:rotConsts">

<array units="cm-1"> 0.925219142 0.175176805 0.163610807 </array>

</property>

<property dictRef="me:vibFreqs">

<array units="cm-1"> 148.644 356.5845 460.3716 780.9087 830.9782 983.4914 1052.7939 1137.8264 1366.0593 1390.2772 1446.2163 1468.6372 1615.3091 3045.8735 3128.1652 3163.3739 3186.2878 </array>

</property>

<property title="ImaginaryFrequency" dictRef="me:imFreqs">

<array units="cm-1"> 576.5907 </array>

</property>

<property dictRef="me:frequenciesScaleFactor">

<scalar>1</scalar>

</property>

<property dictRef="me:symmetryNumber">

<scalar>1</scalar>

</property>

<property dictRef="me:MW">

<scalar units="amu">60</scalar>

</property>

<property dictRef="me:spinMultiplicity">

<scalar>3</scalar>

</property>

</propertyList>

<me:DOSCMethod name="ClassicalRotors"/>

</molecule>

<molecule id="MDIOX">

<atomArray>

<atom id="a1" elementType="C" x3="-0.711005" y3="0.555783" z3="0.012967"/>

<atom id="a2" elementType="H" x3="-1.141679" y3="1.549082" z3="0.034816"/>

<atom id="a3" elementType="H" x3="-2.497705" y3="-0.487454" z3="0.033399"/>

<atom id="a4" elementType="C" x3="-1.422271" y3="-0.561487" z3="0.008612"/>

<atom id="a5" elementType="H" x3="-0.965840" y3="-1.536119" z3="-0.030796"/>

<atom id="a6" elementType="O" x3="0.647526" y3="0.710445" z3="-0.018173"/>

<atom id="a7" elementType="O" x3="1.307633" y3="-0.573770" z3="-0.097132"/>

<atom id="a8" elementType="H" x3="1.763602" y3="-0.584683" z3="0.755546"/>

</atomArray>

<propertyList>

<property dictRef="me:ZPE">

<scalar units="kJ/mol"> -109.934 </scalar>

</property>

<property dictRef="me:vibFreqs">

<array units="cm-1"> 204.3565 385.0632 455.9851 780.9796 839.1231 938.7126 1031.6615 1133.4651 1208.6420 1301.0413 1402.3023 1445.4865 1478.6479 1491.9822 3037.2078 3079.8326 3096.0492 3133.6495 </array>

</property>

<property dictRef="me:frequenciesScaleFactor">

```xml
  <scalar>1</scalar>
 </property>
 <property dictRef="me:symmetryNumber">
  <scalar>1</scalar>
 </property>
 <property dictRef="me:MW">
  <scalar units="amu">60</scalar>
 </property>
 <property dictRef="me:spinMultiplicity">
  <scalar>1</scalar>
 </property>
</propertyList>
<me:DOSCMethod name="ClassicalRotors"/>
 <me:energyTransferModel xsi:type="me:ExponentialDown">
 <me:deltaEDown units="cm-1" derivedFrom="antiC2:deltaEDown" factor="1.0" addand="0.0">300</me:deltaEDown>
</me:energyTransferModel>
</molecule>

<molecule id="He">
<atom elementType="He" />
<propertyList>
 <property dictRef="me:epsilon">
```

<scalar>10.2</scalar>
</property>
<property dictRef="me:sigma">
<scalar>2.55</scalar>
</property>
<property dictRef="me:MW">
<scalar units="amu">4.0</scalar>
</property>
</propertyList>
</molecule>

<molecule id="N2">
<atom elementType="N"/>
<propertyList>
<property dictRef="me:epsilon">
<scalar>423.0</scalar>
</property>
<property dictRef="me:sigma">
<scalar>5.39</scalar>
</property>
<property dictRef="me:MW">
<scalar units="amu">28.0</scalar>

```
  </property>
 </propertyList>
</molecule>

</moleculeList>

<reactionList>
 <reaction id="R1">
  <reactant>
   <molecule ref="antiC2" me:type="modelled" />
  </reactant>
  <product>
   <molecule ref="MDIOX" me:type="modelled" />
  </product>
  <me:MCRCMethod name="SimpleRRKM"/>
  <me:transitionState>
   <molecule ref="TS" me:type="transitionState" />
  </me:transitionState>
 </reaction>
</reactionList>

<me:conditions>
```

```
 <me:bathGas>N2</me:bathGas>
 <me:PTs>

 <me:PTpair me:units="Torr" me:P="760" me:T="298" me:precision= "d" />

 </me:PTs>
</me:conditions>

<me:modelParameters>
 <me:grainSize units="cm-1">50</me:grainSize>
 <me:energyAboveTheTopHill>25.0</me:energyAboveTheTopHill>
</me:modelParameters>

<me:control>
 <me:testDOS />
 <me:printSpeciesProfile />
 <me:testRateConstant />
 <me:printGrainDOS />
 <me:printGrainkfE />
 <me:printGrainkbE />
 <me:eigenvalues>0</me:eigenvalues>
</me:control>
```

</me:mesmer>
